# An empirical model of proton RBE based on the linear correlation between x‐ray and proton radiosensitivity

**DOI:** 10.1002/mp.15850

**Published:** 2022-07-28

**Authors:** David B. Flint, Chase E. Ruff, Scott J. Bright, Pablo Yepes, Qianxia Wang, Mandira Manandhar, Mariam Ben Kacem, Broderick X. Turner, David K. J. Martinus, Simona F. Shaitelman, Gabriel O. Sawakuchi

**Affiliations:** ^1^ Department of Radiation Physics The University of Texas MD Anderson Cancer Center Houston Texas USA; ^2^ Department of Physics and Astronomy Rice University Houston Texas USA; ^3^ The University of Texas MD Anderson Cancer Center UTHealth Graduate School of Biomedical Sciences Houston Texas USA; ^4^ Department of Radiation Oncology The University of Texas MD Anderson Cancer Center Houston Texas USA

**Keywords:** DNA repair, proton therapy, radiosensitivity, relative biological effectiveness

## Abstract

**Background:**

Proton relative biological effectiveness (RBE) is known to depend on physical factors of the proton beam, such as its linear energy transfer (LET), as well as on cell‐line specific biological factors, such as their ability to repair DNA damage. However, in a clinical setting, proton RBE is still considered to have a fixed value of 1.1 despite the existence of several empirical models that can predict proton RBE based on how a cell's survival curve (linear‐quadratic model [LQM]) parameters α and β vary with the LET of the proton beam. Part of the hesitation to incorporate variable RBE models in the clinic is due to the great noise in the biological datasets on which these models are trained, often making it unclear which model, if any, provides sufficiently accurate RBE predictions to warrant a departure from RBE = 1.1.

**Purpose:**

Here, we introduce a novel model of proton RBE based on how a cell's intrinsic radiosensitivity varies with LET, rather than its LQM parameters.

**Methods and materials:**

We performed clonogenic cell survival assays for eight cell lines exposed to 6 MV x‐rays and 1.2, 2.6, or 9.9 keV/µm protons, and combined our measurements with published survival data (*n* = 397 total cell line/LET combinations). We characterized how radiosensitivity metrics of the form D_SF%_, (the dose required to achieve survival fraction [SF], e.g., D_10%_) varied with proton LET, and calculated the Bayesian information criteria associated with different LET‐dependent functions to determine which functions best described the underlying trends. This allowed us to construct a six‐parameter model that predicts cells’ proton survival curves based on the LET dependence of their radiosensitivity, rather than the LET dependence of the LQM parameters themselves. We compared the accuracy of our model to previously established empirical proton RBE models, and implemented our model within a clinical treatment plan evaluation workflow to demonstrate its feasibility in a clinical setting.

**Results:**

Our analyses of the trends in the data show that D_SF%_ is linearly correlated between x‐rays and protons, regardless of the choice of the survival level (e.g., D_10%_, D_37%_, or D_50%_ are similarly correlated), and that the slope and intercept of these correlations vary with proton LET. The model we constructed based on these trends predicts proton RBE within 15%–30% at the 68.3% confidence level and offers a more accurate general description of the experimental data than previously published empirical models. In the context of a clinical treatment plan, our model generally predicted higher RBE‐weighted doses than the other empirical models, with RBE‐weighted doses in the distal portion of the field being up to 50.7% higher than the planned RBE‐weighted doses (RBE = 1.1) to the tumor.

**Conclusions:**

We established a new empirical proton RBE model that is more accurate than previous empirical models, and that predicts much higher RBE values in the distal edge of clinical proton beams.

## 1 INTRODUCTION

The relative biological effectiveness (RBE) of protons varies with physical parameters of the beam, such as its linear energy transfer (LET) and the dose delivered, and biological considerations such as the choice of endpoint, or aspects of cell's intrinsic radiosensitivity, including its photon survival curve parameters.^1^ However, in most clinical scenarios, despite the existence of several models that can predict proton RBE^2^, ^3^, ^4^, ^5^, ^6^, ^7^ and studies demonstrating the feasibility of RBE‐weighted dose‐optimization,^8^ a global RBE value of 1.1 is still typically used for optimizing clinical treatment plans in line with the AAPM's TG256's recommendations.^9^

Several factors contribute to the hesitation to perform RBE‐weighted dose‐optimization in the context of proton therapy treatment planning. First, although many models have been proposed to predict proton RBE, they are often at odds with each other's predictions, and it is unclear which model provides the best description of how RBE varies as a function of proton LET.^10^ Second, the RBE values in proton therapy do not deviate greatly from 1.1 across most of the treatment field,^11^ so accounting for their divergence from 1.1 in treatment planning may not result in appreciably different treatment plans. Third, biological variations from tumor to tumor may be larger than the LET's effect on RBE and not accounting for these biological variations may result in large errors.^12^, ^13^ Fourth, the biological data used to evaluate the different models are extremely noisy, which can mean that the uncertainty in the biological response may be greater than the divergence of RBE from 1.1, rendering it difficult to meaningfully assess the relative accuracy of the different models.^9^ Nevertheless, that TG256 recommends vendors to include proton RBE models in their treatment planning systems^9^ suggests that the need for reliable and easy‐to‐implement proton RBE models is on the horizon.

Current proton RBE models fall broadly into two categories—mechanistic models/mathematical models, for example, the local effect model (LEM),^3^ the microdosimetric kinetic model (MKM),^4^ the repair‐misrepair‐fixation (RMF) model,^5^ and other recently published models^14^, ^15^, ^16^; and empirical models such as the Wedenberg,^2^ McNamara,^6^ Mairani^7^ models, and other models.^17^, ^18^ With respect to the mechanistic models, while these models may offer insights into the underlying processes governing how cell radiosensitivity varies between radiation qualities, they face a major limitation in that they rely on cell‐line‐specific parameters other than the linear‐quadratic model (LQM) survival curve parameters α_x‐ray_ and β_x‐ray_ as input parameters. For example, the LEM requires the radius of the cell nucleus,^3^ the MKM requires the radius of the cell nucleus and the nuclear domain,^4^ and the RMF model uses the radius and density of the cell nucleus^5^ as input parameters. Since these values are rarely reported in cell survival studies, and are generally only known for a handful of very commonly used cell lines, despite the vast amounts of published cell survival data for cells exposed to protons,^1^ it is very difficult to broadly validate the accuracy of the predictions made with these mechanistic models without approximating the unknown cell‐line‐specific parameters. Thus, it remains an open question as to the relative performance of the different mechanistic models in addition to whether they are more or less accurate than their empirical counterparts.

The empirical approaches, on the other hand, are much more suited to validation against the available survival data, since they generally only require α_x‐ray_ and β_x‐ray_ as well as beam quality specifiers, such as LET, as input parameters. Notably, Wedenberg et al.’s model^2^ predicts proton RBE based on a linear dependence of α_proton_/α_x‐ray_ and β_proton_/β_x‐ray_ on proton LET; McNamara et al.’s model^6^ uses a non‐linear function to account for how proton LET modulates a cell's RBE depending on its α_x‐ray_/β_x‐ray_; and Mairani et al.’s model^7^ uses nonlinear functions to model how α_proton_/α_x‐ray_ and β_proton_/β_x‐ray_ vary with proton LET. The empirical models use a pragmatic approach in which the mathematical formulation is chosen simply to describe the trends as accurately as possible without offering insights into the underlying mechanisms governing the trends. Although experimental data to train and validate these models is becoming increasingly more abundant, the amount of variability in these datasets is so great that there is still not much optimism that such approaches will see great improvements going forward.^9^

However, in contrast to previous empirical approaches, here we present a model that is based on trends in how biological endpoints (e.g., the dose resulting in 10% survival, D_10%_, or the surviving fraction after a dose of 2 Gy, SF_2Gy_) vary with LET, whereas previous modeling efforts have focused on describing how the biological parameters themselves (e.g., α or α/β) vary with LET. This difference, although seemingly subtle, confers an important benefit to our approach: estimating endpoints like D_10%_ precisely is much less difficult than estimating α or β from a cell survival curve since α and β tend to have negative covariances. This means that for the same training dataset, the trends we model are inherently less noisy than the trends in α and β themselves, mitigating one of the great challenges in modeling the underlying trends in the data in the first place.

This new model of proton RBE is based on the linear correlation between cell radiosensitivity to ions and x‐rays, first reported by Suzuki et al.^19^ for carbon ions. As we show in this work, this strong correlation holds for protons as well, and across a wide range of biological endpoints. By modeling the LET trends in these correlations we constructed a model that predicts proton RBE.

## 2 MATERIALS AND METHODS

### 2.1 In‐house survival experiments

We performed clonogenic assays to quantify cell survival after irradiation in eight human cancer cell lines (H460 and H1299 [non‐small cell lung cancer]; M059K and M059J [glioblastoma]; BxPC3 [pancreatic adenocarcinoma]; and HT1080, HT1080‐shDNA‐PKcs, and HT1080‐shRAD51^IND^ [fibrosarcoma]). Cells were exposed to 6 MV x‐rays and protons with dose‐weighted LET values of 1.2, 2.6, or 9.9 keV/µm. Further details on these cell lines, irradiation conditions, and how the data were analyzed are published elsewhere.^12^, ^13^ Notably, these cell lines included cells deficient in the DNA repair proteins DNA‐PKcs (M059J and HT1080‐shDNA‐PKcs) and Rad51 (HT1080‐shRAD51^IND^), the inclusion of which did not perturb the trends we observed.

### 2.2 Compilation of training dataset

As a training dataset, we combined our in‐house survival experiments with published survival data from Liu et al.’s study of lung cancer cell lines,^20^ the PIDE Database version 3.2,^21^, ^22^ and the data summarized in Paganetti et al.’s review.^1^ Some experiments summarized in the particle irradiation data ensemble (PIDE) were also summarized in Paganetti's review article, but since these authors used slightly different methods to aggregate the data, the reported results are not identical. For these cases, in an effort to maintain as much consistency as possible, we used the data reported in the PIDE, because this single source contains data across a large number of experiments that were all reanalyzed using similar methods. The total number of paired survival experiments (cell line/LET combinations) within this combined dataset was *n* = 471.

From this combined database, we filtered out data that rendered our analyses untrustworthy or impossible as follows. We excluded data where β ≤ 0 (*n* = 42 paired datasets) for three reasons: (i) negative β values are often non‐physical, resulting often from fitting noise (but mostly linear) survival data to the LQM without any constraints; (ii) for all survival curves we calculated the mean inactivation dose and the L2 norm between the predicted and measured survival curves, and negative β values cause these integrals to diverge; and (iii) null β values cause the ratio of α/β to diverge, which is an important quantity in many other empirical models. We excluded data where the cells were exposed under hypoxic conditions (*n* = 7 paired datasets). This is because under hypoxic conditions, the RBE values are considerably larger than those in normoxic conditions due to the additional effect of the oxygen enhancement ratio (OER), and these considerably larger RBE values may bias our fits (and goodness‐of‐fit assessments) toward a relatively small subset of the training data. Also, under hypoxic conditions, cells are often extremely radioresistant, and given that the extent to which we can use the LQM to model cell survival above 15 Gy is unclear,^23^ estimating important biological endpoints (e.g., D_10%_) from the predicted α and β values under these conditions may have considerably greater uncertainty than can be accounted for. For this same reason, we also excluded data for the cell line HTB140 (*n* = 8 paired datasets), whose D_10%_ values are >40 Gy. We further excluded data where the survival was assessed by viability assays as opposed to clonogenic survival assays (*n* = 7 datasets) to ensure more consistency between experiments. Additionally, similarly to Mairani et al.,^7^ we further excluded data for proton LET values >37.8 keV/µm (*n* = 2 paired datasets), because these LET values are much higher than those of clinical relevance, and the resulting large RBE values may bias our goodness‐of‐fit assessments. Finally, we excluded datasets for which the reference photon radiation source was listed with a nominal energy <200 kVp (*n* = 19 paired datasets). This was done as very low‐energy x‐ray sources may be more biologically effective than high energy photon sources (e.g., 6 MV x‐rays or Co‐60), which comprise the majority of the reference photon data. In total, this resulted in us including 397 cell line/LET combinations in our training dataset (81% of the available data). Note that some data were excluded from our dataset for failing multiple criteria.

More details about the data included in our training dataset including their survival curve parameters can be found in Note S1.

### 2.3 Statistical analyses

All statistical analyses were performed in MATLAB 2020 (Mathworks, Natick, MA) and Graph Pad Prism 7 (Graph Pad, San Diego, CA). For our radiosensitivity metrics (e.g., D_10%_), the error bars represent the standard error propagated from the fitted survival curve parameters (α and β), including their covariance, to the radiosensitivity parameters estimated from them. The confidence intervals in the measured survival curves represent the uncertainty from our fit parameters, including their covariance, propagated into our predictive function, calculating the 95% confidence intervals as ±1.96 times the standard error of the prediction. Additional details related to fitting our model are given in Note S2.

### 2.4 Assessment of linear correlations between radiosensitivity to protons and x‐rays

To assess the correlations between radiosensitivity to protons and x‐rays, we computed the following radiosensitivity metrics for each radiation quality: D_5%_, D_10%_, D_20%_, D_37%_, D_50%_, and SF_2Gy_. For each metric, we quantified the correlation between radiation qualities via the Pearson correlation coefficient (r).

### 2.5 Parameterization of the LET dependence of the linear correlations

To assess the LET dependence of the linear correlations we noted that since radiosensitivity parameters such as D_10%_ are linearly correlated, we can write a general expression of the form:

(1) $$D_{10\%,\mathrm{proton}}=D_{10\%,x-\mathrm{ray}}\cdot \mathrm{slope}\left(\mathrm{LET}\right)+\mathrm{inte}\mathrm{rcept}\left(\mathrm{LET}\right)$$

describing the slopes’ and intercepts’ proton LET dependence. Using this general expression, the faithfulness of any LET‐dependent functions describing the slope or intercept of the linear correlations can be assessed using paired survival data which need not share a common proton LET. In this way, we assessed the accuracy of selected several candidate slope and intercept functions against the whole training dataset, calculating the Bayesian information criterion (BIC)^24^ associated with each function's fit of the data to quantify which parameterization best described the underlying trends without the inclusion of unnecessary free parameters. These functions are summarized in Table 1 (see Note S3 for more details).

**TABLE 1:** Candidate slope and intercept functions with free parameters c, f, g, h, k, m, p, q, and s, chosen to assess the linear energy transfer (LET) dependence of the slope and intercept of the linear correlation between proton and x‐ray radiosensitivity. The functions were chosen to either increase (intercept) or decrease (slope) with increasing LET, but with additional functions chosen to allow for non‐monotonic behavior

Slope		Intercept	
Function	Behavior	Function	Behavior
$c\cdot e^{-f\cdot \mathrm{LET}}$	Slope decreases exponentially with LET, asymptotically approaching zero.	*m*	Intercept has the same constant value for all LET values.
$c\cdot e^{-f\cdot \mathrm{LET}}+g$	Slope decreases exponentially with LET and asymptotes to a non‐zero value.	p·LET	Intercept increases linearly with LET.
$c\cdot e^{-f\cdot LET-h\cdot LET^{2}}+g$	Slope follows a Gaussian dependence on LET, allowing non‐monotonic behavior but decreasing exponentially for high LET values.	m+p·LET	Intercept increases linearly with LET with a constant offset.
$\left(c+h\cdot LET\right)\cdot e^{-f\cdot LET}+g$	Slope depends on the product of an increasing linear and decreasing exponential dependence on LET, allowing for non‐monotonic behavior that ultimately decreases exponentially for high LET values.	$m+p\cdot LET+\;q\cdot LET^{2}\;$	Intercept increases quadratically with LET, allowing for non‐monotonic behavior.
$c\cdot ln\left(LET-h\right)\cdot e^{-f\cdot LET}+g$	Slope depends on the product of an increasing logarithmic and decreasing exponential dependence on LET, allowing for non‐monotonic behavior that ultimately decreases exponentially for high LET values.	$q\cdot e^{s\cdot \mathrm{LET}}$	Intercept increases exponentially with LET.
$\frac{c}{\Gamma (f\cdot LET+h+1)}+g$	Slope decreases via an inverse gamma dependence on LET—this is motivated by the Poisson‐like distribution described below, but with fewer free parameters.	$q\cdot e^{s\cdot \mathrm{LET}}+m$	Intercept increases exponentially with LET, beginning at a small positive value.
$\frac{c\;k^{f\cdot (LET-h)}\cdot e^{-k}}{\Gamma (f\cdot (LET-h)+1)}+g$	Slope follows a Poisson‐like (functionally similar, but continuous) LET dependence, allowing for non‐monotonic LET dependence while being parameterized by a function of particular relevance to radiation biology.	$q\cdot e^{-s\cdot LET}+p\cdot LET+m$	Intercept initially decreases exponentially with LET before increasing linearly at higher LET values, allowing for non‐monotonic behavior.

### 2.6 General model of proton RBE

As many endpoints’ responses can be modeled by predictive functions similar to Equation (1) (e.g., D_5%_, D_20%_, D_37%_, D_50%_, and SF_2Gy_), a set of endpoints can be predicted that allows for the α_proton_ and β_proton_ values to be determined from fitting the predicted data to the LQM.

However, explicitly performing a nonlinear fit (which can be computationally expensive) is unnecessary, as our formalism can be reduced into an expression for α_proton_ and β_proton_ that requires only the LET of the proton beam and the α_x‐ray_ and β_x‐ray_ values for the cell line of interest as input parameters, as well as the model parameters associated with whatever endpoints, i, are selected. The following general expressions give α_proton_ and β_proton_ in terms of the D_SF_ values given by Equation (1) for whatever survival endpoints, SF, are selected (see Note S4 for more details):

(2) $$\begin{array}{c} \alpha_{\mathrm{proton}}=\;\frac{\sum_{i}D_{\mathrm{SF},i}^{4}\sum_{i}D_{\mathrm{SF},i}\log\left(SF_{i}\right)-\sum_{i}\;D_{\mathrm{SF},i}^{3}\sum_{i}D_{\mathrm{SF},i}^{2}\log\left(SF_{i}\right)}{\sum_{i}D_{\mathrm{SF},i}^{3}\sum_{i}\;D_{\mathrm{SF},i}^{3}-\sum_{i}D_{\mathrm{SF},i}^{2}\;\sum_{i}D_{\mathrm{SF},i}^{4}}\; \end{array}$$

and

(3) $$\begin{array}{c} \beta_{\mathrm{proton}}=\;\frac{\sum_{i}D_{\mathrm{SF},i}^{2}\sum_{i}D_{\mathrm{SF},i}^{2}\log\left(SF_{i}\right)-\sum_{i}D_{\mathrm{SF},i}^{3}\sum_{i}D_{\mathrm{SF},i}\log\left(SF_{i}\right)}{\sum_{i}D_{\mathrm{SF},i}^{3}\sum_{i}\;D_{\mathrm{SF},i}^{3}-\sum_{i}D_{\mathrm{SF},i}^{2}\;\sum_{i}D_{\mathrm{SF},i}^{4}}\; \end{array}$$

### 2.7 Constraining α ≥ 0 and β ≥ 0

Equations (2) and (3) minimize the distance between the set of predicted endpoints and the survival curve described by α_proton_ and β_proton_. However, when α and β are close to zero, occasionally their values will be found to be negative. This becomes an issue as we wish to compute the distance between the predicted and measured curves using the L2 norm, and having a negative β value will result in a divergent integral. Thus, for cases where α or β were predicted to be negative, we imposed upon them a null value, and solved for α or β alone as follows:

(4) $$\begin{array}{c} \alpha_{\mathrm{proton}\;\left(\beta =0\right)}=\;-\frac{\sum_{i}D_{\mathrm{SF},i}\log\left(SF_{i}\right)}{\sum_{i}D_{\mathrm{SF},i}^{2}\;} \end{array}$$

and

(5) $$\begin{array}{c} \beta_{\mathrm{proton}\;\left(\alpha =0\right)}=\;-\frac{\sum_{i}D_{\mathrm{SF},i}^{2}\log\left(SF_{i}\right)}{\sum_{i}D_{\mathrm{SF},i}^{4}}\; \end{array}$$

This is analogous to fitting survival data to the LQM under the common constraint that α ≥ 0 and β ≥ 0.

### 2.8 Choice of endpoints to incorporate in our model

Equations (1)‐(5) are valid for any number of arbitrarily chosen endpoints. The use of additional endpoints will improve the accuracy of the predicted α_proton_ and β_proton_ values at the cost of increased complexity of the model. To weigh how much more faithfully the model reproduces the data against the informational cost of including additional parameters, we created predictive functions for each of D_5%_, D_10%_, D_20%_, D_37%_, D_50%_, and SF_2Gy_, and calculated the BIC associated with each possible combination of endpoints used to construct a model, using the sum of L2 norms between the predicted and measured survival curves (normalized by the mean inactivation dose) as the distance metric. Further details of these analyses are given in Note S5.

### 2.9 Assessment of model accuracy

To assess the general accuracy of our six‐parameter model, we performed leave‐one‐out cross‐validation across the whole training dataset, excluding each datapoint individually, retraining the model on the remaining data, and predicting the response of the excluded datapoint. At the 0.5, 1, 2, and 5 dose levels, we calculated the deviations in our model's RBE predictions and bootstrapped them to estimate prediction intervals associated with our model.

### 2.10 Comparison to other empirical proton RBE models

We compared the accuracy of our model with the empirical models proposed by Wedenberg et al.,^2^ McNamara et al.,^6^ and Mairani et al.^7^ We retrained these models using our training dataset to ensure that the success of any particular model did not depend on the data used in its creation. This retraining was performed in two ways: (i) by minimizing the residual sum of squares (RSS) between the predicted and measured RBE values at the 2 Gy dose level and (ii) by minimizing the L2 norms between the predicted and measured survival curves normalized by the mean inactivation dose (as described in Note S5). This first method produces models optimized to predict the RBE for a proton dose of 2 Gy which is approximately the dose per fraction delivered commonly in clinical settings. The second method, which is analogous to minimizing the sum‐of‐squares distance between the predicted and measured survival curves across all dose levels, produces the models that are the most robust across all dose levels. We computed two metrics to quantify the goodness‐of‐fit of the models: (i) the BIC associated with the fit for both minimizations and (ii) the reduced chi‐squared statistic, χ2/ν, when minimizing the RSS. These metrics both weigh the accuracy of the models’ predictions against the number of parameters that are used in their creation, with the BIC using a much greater penalty for the inclusion of additional free parameters. The explicit parameterizations used for the Wedenberg et al.,^2^ McNamara et al.,^6^ and Mairani et al.^7^ models are given in Note S6 along with the parameter values found.

Notably, the models by Wedenberg et al.^2^ and McNamara et al.^6^ were originally trained on data spanning different ranges of LET values, considering only LET values up to 30 and 20 keV/μm, respectively. To ensure our analyses were not biased toward the inclusion of very high LET data, we compared the models’ performance again, but after filtering the data to these LET ranges (see Note S7). McNamara et al.^6^ further excluded data where (α/β)_x‐rays_ > 30 Gy; however, because neither Wedenberg et al.^2^ nor Mairani et al.^7^ made this distinction despite their models containing (α/β)_x‐rays_ as a parameter, and because our model gives no special importance to α/β, we did not exclude these data from our dataset.

### 2.11 Implementation in clinical plan evaluation workflow

Expressing our model via Equations (2)‐(5) allows it to be incorporated into the workflow to evaluate a clinical treatment plan alongside other empirical RBE models. To demonstrate this, we implemented a version of our model within a validated^25^ GPU‐implemented^26^ track‐repeating fast Monte Carlo (FMC) algorithm^27^ to rapidly score the dose and LET distributions in voxelized computed tomography (CT) datasets from proton therapy treatment fields. The FMC infrastructure then allows these data to be exported to and displayed in a treatment planning system. In this manner, we retrospectively calculated 3D RBE‐weighted dose distributions for a patient treatment plan according to our model's predictions alongside those of the Wedenberg et al.^2^ and McNamara et al.^6^ models. The plan we selected was for a patient who received a 54 Gy_RBE_ (27 fractions) course of intensity modulated proton therapy delivered from four beam directions for cancer of the anal canal.

## 3 RESULTS

### 3.1 The linear correlation between x‐ray and proton radiosensitivity

**FIGURE 1:**
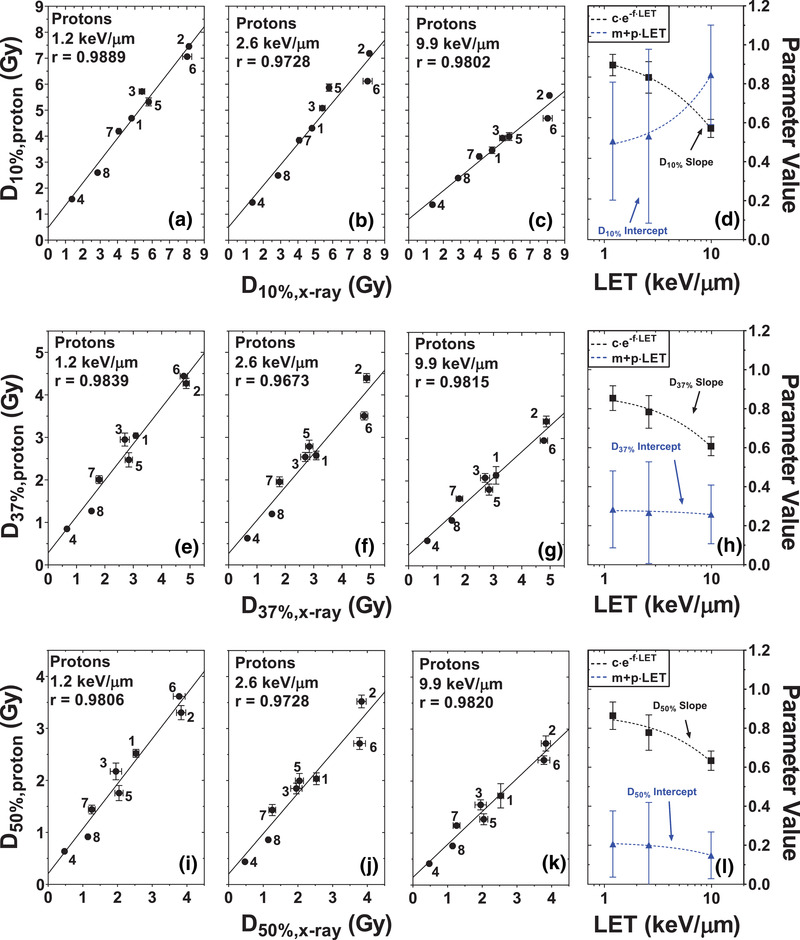
Linear correlation between proton and x‐ray radiosensitivity for the radiosensitivity, parameterized by the dose required to reduce cell survival to 10%, D_10%_ (a–d); 37%, D_37%_ (e–h); and 50%, D_50%_ (i–l), for cells exposed to 6 MV x‐rays and protons with dose‐weighted linear energy transfer (LET) values of 1.2 keV/µm (a, e, i), 2.6 keV/µm (b, f, j) or 9.9 keV/µm (c, g, k). The values of the correlations’ slopes (black) and intercepts (blue) are given in panels (d), (h), and (l), with the dashed lines showing exponential fits of the data. Numbers indicate the cell lines as follows: 1 = H460, 2 = H1299, 3 = M059K, 4 = M059J, 5 = BxPC3, 6 = HT1080, 7 = HT1080‐shRad51^IND^, and 8 = HT1080‐shDNAPKcs. Trends for other radiosensitivity parameters are given in Note S7

Similar to Suzuki et al.’s^19^ observation that D_10%_ is linearly correlated between x‐rays and carbon ions, our in‐house data show that this is also true for protons and that this correlation holds regardless of the choice of endpoint selected to characterize cell radiosensitivity (Figure 1). In addition to this, regardless of the choice of radiosensitivity endpoint, the slope of these correlations decreases for increasing particle LET (Figure 1d,h,l) and the intercept may also vary with LET. However, because we acquired data at only three distinct LET values, it is difficult to justify any particular parameterization of the slopes’ and intercepts’ LET dependence based solely on these data.

### 3.2 Choice of LET‐dependent function to parameterize the slope and intercept of the linear correlations

To better assess these LET dependencies of the slope and intercept, we used Equation (1) to test the accuracy of several *slope(LET)* and *intercept(LET)* functions (Table 1) against the whole training dataset. For every combination of slope and intercept function, we created a predictive function similar to Equation (1) to predict each of D_5%_, D_10%_, D_20%_, D_37%_, D_50%_, and SF_2Gy_, fitting the resulting sets of free parameters to the training dataset using MATLAB's *lsqnonlin* function to minimize the relative square distance between the predicted and measured endpoint. We calculated the BIC^24^ associated with each fit to determine which parameterizations best reproduced the underlying trends in the data. Table 2 shows these data for the endpoint D_10%_ (data for the other endpoints can be found in Note S3). From these data, the function of the form:

(6) $$\begin{array}{c} \mathrm{Slope}\left(\mathrm{LET}\right)=c\cdot e^{-f\cdot \mathrm{LET}} \end{array}$$

minimizes the BIC values, and thus is the best choice of function to model the slope's LET dependence.

**TABLE 2:** Bayesian information criteria (BIC) values determined by fitting the function created by combining the candidate slope and intercept functions to the training dataset's D_10%_ values. Each cell corresponds to the BIC value associated with a given slope and intercept function combination. Green cells indicate smaller BIC values (better performing functions) while red cells indicate larger BIC values (poorer performing functions). BIC values for D_5%_, D_20%_, D_37%_, D_50%_, and SF_2Gy_ can be found in Note S2

Bayesian information criterion (BIC) value associated with the function predicting D_10%_							
Intercept							
slope	*m*	p·LET	m+p·LET	$m+p\cdot LET+q\cdot LET^{2}$	$q\cdot e^{s\cdot \mathrm{LET}}$	$q\cdot e^{s\cdot \mathrm{LET}}+m$	$q\cdot e^{s\cdot LET}+\;p\cdot LET+m$
$c\cdot e^{-f\cdot \mathrm{LET}}$	−155.6	−146.6	−153.4	−147.4	−152.3	−146.4	−141.4
$c\cdot e^{-f\cdot \mathrm{LET}}+g$	−149.9	−140.6	−147.4	−141.4	−146.4	−140.4	−135.4
$c\cdot e^{-f\cdot LET-h\cdot LET^{2}}+g$	−144.1	−142.5	−142.7	−136.7	−142.0	−136.1	−130.7
$\left(c+h\cdot LET\right)\cdot e^{-f\cdot LET}+g$	−144.0	−140.8	−142.4	−136.5	−141.5	−135.5	−130.5
$c\cdot ln\left(LET-h\right)\cdot e^{-f\cdot LET}+g$	−144.0	−139.6	−142.2	−136.2	−141.2	−135.9	−130.2
$\frac{c}{\Gamma (f\cdot LET+h+1)}+g$	−144.1	−141.9	−142.6	−136.6	−141.8	−135.8	−130.7
$\frac{c\;\lambda^{f\cdot (LET-h)}\cdot e^{-k}}{\Gamma (f\cdot (LET-h)+1)}+g$	−138.1	−136.4	−136.7	−130.7	−15.2	−130.0	−127.4

For the intercept, there are several candidate functions that provide comparable descriptions of the data, namely, the constant function, the linear function, or the exponential function with no vertical offset (Table 1). As is described in the sections below, further comparisons made by combining multiple endpoints into a single predictive model suggest that the linear function with no offset offers a suitable description of the data when predicting α_proton_ and β_proton_ using multiple endpoints:

(7) $$\begin{array}{c} \mathrm{Inte}\mathrm{rcept}\left(\mathrm{LET}\right)=p\cdot \mathrm{LET} \end{array}$$

### 3.3 Predicting survival curves under our formalism

**FIGURE 2:**
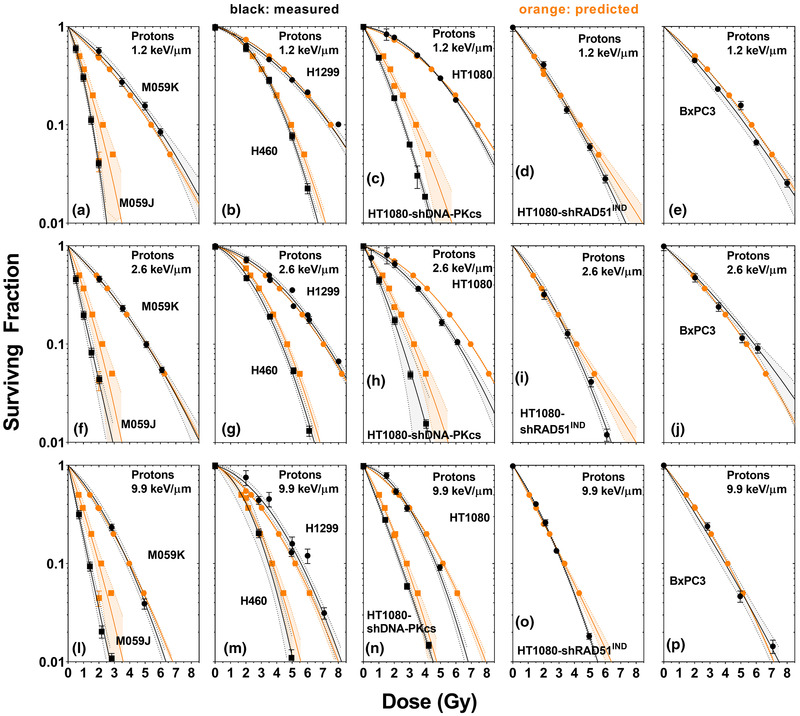
Predicted (orange, with 95% confidence interval) and measured (black, with 95% confidence interval) survival curve for M059K and M059J cells (a, f, k), H460 and H1299 cells (b, m, g), HT1080 and HT1080‐shDNA‐PKcs cells (c, h, n), HT1080‐shRad51^IND^ cells (d, i, o), and BxPC‐3 cells (e, j, p) exposed to protons with dose‐weighted linear energy transfer (LET) values of 1.2 (a–e), 2.6 (f–j), and 9.9 keV/µm (l–p). Shaded areas are 95% confidence intervals. The curves were predicted after creating predictive functions for D_5%_, D_10%_, D_20%_, D_37%_, D_50%_, and SF_2Gy_ and fitting these functions to the combined data, excluding the data to be predicted. The confidence intervals were calculated based on the uncertainty in the predicted α and β values, which were determined from the residuals of the fit and the associated covariance matrix. These were determined by inverse‐variance‐weighted fits of the experimental data to the linear‐quadratic model (LQM) in GraphPad prism, or by fitting and the predicted endpoints to the LQM using MATLAB's lsqnonlin function

Since a wide range of biological endpoints can be predicted by equations similar to Equation (1) (e.g., D_5%,_ D_10%,_ D_20%,_ D_37%,_ D_50%_, and SF_2Gy_), the survival curve (α_proton_ and β_proton_) can be estimated from the curve that best describes the predicted endpoints, that is, via Equations (2)‐(5). To demonstrate this, we trained functions similar to Equation (1) to predict each of the following endpoints: D_5%,_ D_10%,_ D_20%,_ D_37%,_ D_50%_, and SF_2Gy_. We then used these functions to predict these radiosensitivity endpoints for the survival curves we collected, after excluding each predicted curve when training the functions. We then fit these predictions to the LQM to determine the predicted α_proton_ and β_proton_ values, with the confidence intervals on the predicted curves being determined from the fitting uncertainties in α_proton_ and β_proton_. These predictions are shown in Figure 2. Note that the predictions for the SF_2Gy_ endpoint were much less accurate and much less reliable than the prediction of endpoints of the form D_N%, proton_. Consequently, the relative uncertainties in predicting SF_2Gy_ are much larger than the other endpoints, and there is much more scatter of this particular endpoint around the estimated trendline than the other predicted endpoints.

### 3.4 Optimal choice endpoints to use for our model

Since Equations (2)‐(5) are compatible with any subset of endpoints, it is not obvious how many and which endpoints are needed to make sufficiently accurate predictions. To address this question, for every combination of two to six endpoints, we predicted the α_proton_ and β_proton_ values according to Equations (2)‐(6), and calculated the BIC value associated with the fit of the training data, using the L2 norms normalized by the mean inactivation dose as the distance metric. Since it was unclear which function best described the LET dependence of the intercepts from our initial fitting of the candidate functions (Table 2), we performed these analyses in parallel using several possible parameterizations of the intercept's LET dependence (constant, linear, and exponential) to determine which parameterization yielded the best description of the data in the context of predicting α_proton_ and β_proton_. In general, we saw that regardless of parameterization, the best description of the data were the ones using the fewest parameters, that is, only two endpoints and either the constant intercept, or a linearly increasing intercept with no vertical offset (Table 3).

**TABLE 3:** Bayesian information criteria (BIC) values associated with different formulations of our model created by combining the predictive functions of two endpoints among D_5%_, D_10%_, D_20%_, D_37%_, D_50%_, and SF_2Gy_. Values reported are the smallest BIC value among combinations of a given number of endpoints used to create the model

Minimum BIC values across combinations					
Endpoints used					
Intercept parameterization	**2**	**3**	**4**	**5**	**6**
*m*	−736.3	−714.4	−692.0	−670.8	−650.4
p·LET	−731.0	−714.9	−694.5	−673.6	−652.4
m+p·LET	−727.6	−701.5	−674.6	−648.3	−622.5
$q\;e^{s\cdot LET}$	−726.5	−699.9	−672.7	−646.1	−620.2

When looking at the specific combinations of 2‐endpoint functions, we noted that the specific choice of endpoints that minimize the BIC tends to favor lower survival levels, with the combinations incorporating D_5%_ yielding the lowest BIC values (Table 4). Comparing the different intercept parameterizations, when two low‐survival levels are chosen, a constant tends to be the best parameterization, but when higher survival levels are chosen, the linear function offers better predictive power (Table 4).

**TABLE 4:** Bayesian information criteria (BIC) values associated with specific 2‐endpoint combinations of our model for the endpoints D_5%_, D_10%_, D_20%_, D_37%_, D_50%_, and SF_2Gy_, parameterizing the intercept with either a constant or linear function. Redundant combinations are omitted for ease of interpretation

**BIC values**										
**Intercept parameterizations**	Intercept(LET)=m					Intercept(LET)=p·LET				
**Endpoints**	**D_10%_**	**D_20%_**	**D_37%_**	**D_50%_**	**SF_2Gy_**	**D_10%_**	**D_20%_**	**D_37%_**	**D_50%_**	**SF_2Gy_**
**D_5%_**	−736.3	−731.4	−723.2	−716.1	−664.3	−731.0	−729.0	−725.2	−721.4	−678.9
**D_10%_**		−726.9	−719.3	−713.0	−645.2		−726.3	−721.9	−718.1	−606.7
**D_20%_**			−714.0	−708.6	−657.7			−717.5	−713.4	−656.4
**D_37%_**				−702.2	−704.2				−706.3	−711.7
**D_50%_**					−685.9					−688.3

As we wished our model to be the most robust across dose and survival levels, we wished to incorporate both a lower and a higher survival endpoint into the final parameterization so that the survival curve estimated from them would not be biased toward lower or higher doses. To achieve this, we selected D_5%_ and D_37%_ as the endpoints to use in our formalism as they would also allow us to simplify our final model's construction as described below. Then, as the linear function with no vertical offset (Equation 7) results in the smaller BIC value when estimating α_proton_ and β_proton_ using the endpoints D_5%_ and D_37%_ (Table 4), we used this intercept parameterization in our model.

### 3.5 Final model parameterization

The resulting parameterization encoded into our model was (e.g., for D_37%_):

(8) $$\begin{array}{c} D_{37\%,\mathrm{proton}}=c\cdot e^{-f\cdot \mathrm{LET}}\cdot D_{37\%,x-\mathrm{rays}}+p\cdot \mathrm{LET} \end{array}$$

with free parameters *c*, *f*, and *p*. Our model's final construction incorporated two endpoints, SF_1_ = e^–1^ (∼37%) and SF_2_ = e^–3^ (∼5%) as those specific choices simplify Equations (2)‐(5) by removing the logarithmic terms. This resulted in a six‐parameter model which reduces to the following expressions predicting α_proton_ and β_proton_:

(9a) $$\begin{array}{ccc} & & \alpha_{\mathrm{proton}}=\\ & & \frac{\left(D_{e^{-1}}^{3}+D_{e^{-3}}^{3}\right)\left(D_{e^{-1}}^{2}+3D_{e^{-3}}^{2}\right)-\left(D_{e^{-1}}^{4}+D_{e^{-3}}^{4}\right)\left(D_{e^{-1}}+3D_{e^{-3}}\right)}{{\left(D_{e^{-1}}^{3}+D_{e^{-3}}^{3}\right)}^{2}-\left(D_{e^{-1}}^{2}+D_{e^{-3}}^{2}\right)\left(D_{e^{-1}}^{4}+D_{e^{-3}}^{4}\right)} \end{array}$$

or

(9b) $$\begin{array}{c} \alpha_{\mathrm{proton}\;\left(\beta =0\right)}=\;\frac{D_{e^{-1}}+\;3\;D_{e^{-3}}}{D_{e^{-1}}^{2}+D_{e^{-3}}^{2}} \end{array}$$

and

(10a) $$\begin{array}{ccc} & & \beta_{\mathrm{proton}}=\\ & & \frac{\left(D_{e^{-1}}^{3}+D_{e^{-3}}^{3}\right)\left(D_{e^{-1}}+3D_{e^{-3}}\right)-\left(D_{e^{-1}}^{2}+D_{e^{-3}}^{2}\right)\left(D_{e^{-1}}^{2}+3D_{e^{-3}}^{2}\right)}{{\left(D_{e^{-1}}^{3}+D_{e^{-3}}^{3}\right)}^{2}-\left(D_{e^{-1}}^{2}+D_{e^{-3}}^{2}\right)\left(D_{e^{-1}}^{4}+D_{e^{-3}}^{4}\right)} \end{array}$$

or

(10b) $$\begin{array}{c} \beta_{proton\;\left(\alpha =0\right)}=\frac{D_{e^{-1}}^{2}+3\;D_{e^{-3}}^{2}}{D_{e^{-1}}^{4}+D_{e^{-3}}^{4}},\; \end{array}$$

where the D values are given by equations similar to Equation (8), with free parameters, *c*_1_, *f*_1_, and *g*_1_, associated with SF_1_; and *c*_2_, *f*_2_, and *g*_2_ associated with SF_2_. To determine our model's free parameter values, we fit these functions to our training dataset, minimizing the L2 norms between the predicted and measured survival curves (normalized by the mean inactivation dose) (as described in Note S5). The parameters resulting from this fit are given in Table 5.

**TABLE 5:** Our model's free parameter values, their uncertainties, and their covariances when minimizing the L2 norm between the predicted and measured curves

**Parameter**	**Value**	**Uncertainty**
c1	1.041543E+00	2.439889E‐02
f1	4.708586E‐02	7.931656E‐03
p1	2.376115E‐02	9.021992E‐03
c2	1.045578E+00	5.593340E‐02
f2	3.213278E‐02	1.341988E‐02
p2	6.550494E‐02	3.257196E‐02
cov_c1,f1_	1.072615E‐04	N/A
cov_c1,p1_	6.169650E‐05	N/A
cov_c1,c2_	1.783393E‐04	N/A
cov_c1,f2_	−2.609667E‐05	N/A
cov_c1,p2_	−8.508367E‐05	N/A
cov_f1,p1_	6.242400E‐05	N/A
cov_f1,c2_	−4.298168E‐05	N/A
cov_f1,f2_	−2.548386E‐05	N/A
cov_f1,p2_	−6.796549E‐05	N/A
cov_p1,c2_	−6.503621E‐05	N/A
cov_p1,f2_	−2.627735E‐05	N/A
cov_p1,p2_	−7.880709E‐05	N/A
cov_c2,f2_	3.983314E‐04	N/A
cov_c2,p2_	6.113882E‐04	N/A
cov_f2,p2_	4.084385E‐04	N/A

### 3.6 Accuracy of our six‐parameter model

**FIGURE 3:**
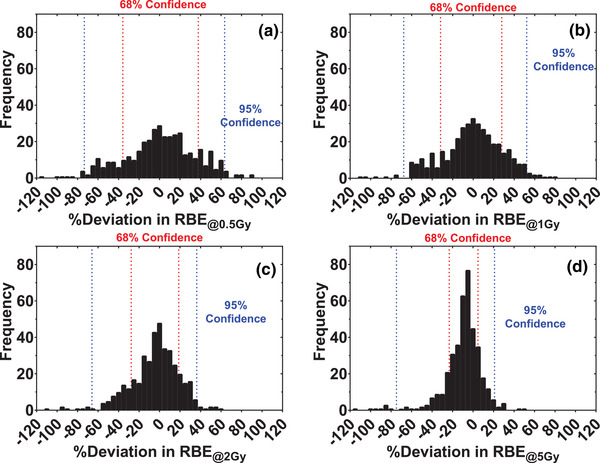
Frequency distribution of the percent deviations between the measured relative biological effectiveness (RBE) values and those predicted by our model trained on the data excluding the predicted point for RBE defined at the (a) 0.5 Gy, (b) 1 Gy, (c) 2 Gy, and (d) 5 Gy dose levels. The red and blue lines show asymmetric 68% and 95% confidence intervals determined by bootstrapping

To quantify the accuracy of our model, we performed leave‐one‐out cross‐validation on the training data to estimate our model's prediction intervals (Figure 3). The approximate range of the 68.3% prediction intervals were on the order of ∼15%–30%, which suggests our model predicts proton RBE within ±15%–30% at the 68.3% confidence level. However, note that our model's accuracy depends strongly on the dose level selected, yielding smaller confidence intervals for higher dose levels than lower dose levels (Figure 3). The accuracy of our model for different dose/survival levels is tabulated in Note S9.

### 3.7 Performance relative to other RBE models

To quantify the relative performance of our model compared to other RBE models we calculated the BIC and χ2/ν values associated with each model's description of the in vitro training dataset. As shown in Table 6, among the Wedenberg et al.,^2^ McNamara et al.,^6^ or Mairani et al.^7^ models, our model yields the smallest goodness‐of‐fit metrics to the training data, regardless of metric or minimization performed, implying that our model provides the most accurate description of the dataset. Furthermore, the considerably smaller BIC values our model yields (which contain a very large penalty for the inclusion of additional free parameters), implies that this is not a result of overfitting. Finally, even when the LET range investigated was constrained to those used in the original works by Wedenberg et al.^2^ (<30 keV/µm) and McNamara et al.^6^ (<20 keV/µm), our model consistently provided the most accurate description of the experimental data (see Note S7 for details). Thus, we believe that among the tested models, our model provides the most robust description of proton RBE for an arbitrarily selected condition.

**TABLE 6:** The reduced chi‐squared statistic (χ2/µ), and Bayesian information criteria (BIC) as goodness‐of‐fit metrics for different models fit to our training dataset, minimizing either the residual sum‐of‐squares in the relative biological effectiveness (RBE) for a proton dose of 2 Gy (RBE_2Gy_), or the L2 norm between the predicted and measured survival curves

**Model**	**Χ^2^/ν (RBE_2Gy_)**	**BIC (RBE_2Gy_)**	**BIC (L2 norm)**
Wedenberg et al.^2^	0.0802	263.5	−719.4
McNamara et al.^6^	0.0731	247.7	−707.9
Mairani et al.^7^	0.0715	261.1	−696.1
Our model	0.0689	239.7	−739.5

### 3.8 Implementation in clinical treatment plan evaluation workflow

**FIGURE 4:**
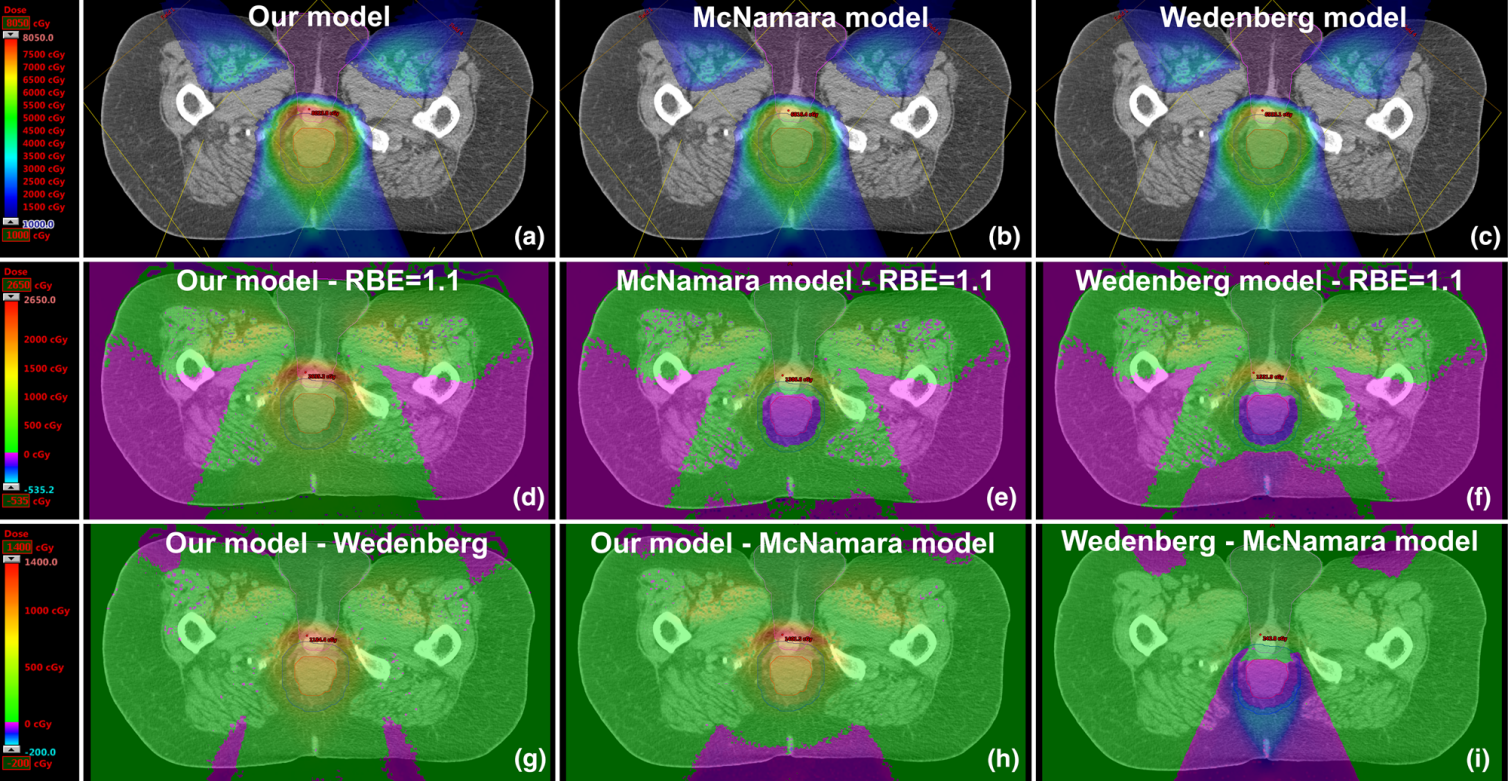
Fast Monte Carlo calculated relative biological effectiveness (RBE) ‐weighted dose distributions shown in the Eclipse treatment planning system, calculated retrospectively for a patient treated with protons for (a) our model, (b) McNamara et al.^6^ model, and (c) Wedenberg et al.^2^ model. The red, navy, and magenta contour lines indicate the gross tumor volume (GTV), the PTV54, and the genitalia, respectively. The arithmetic difference between these models and the assumption that RBE = 1.1 is shown in panels (d–f). The arithmetic difference between our model and the Wedenberg and McNamara models is shown in panels (g) and (h), while the difference between the Wedenberg et al.^2^ and McNamara et al.^6^ models is shown in panel (i)

As a proof‐of‐principle, we implemented our model within a clinical treatment plan evaluation workflow to calculate the RBE‐weighted doses associated with a patient treatment plan (Figure 4). Similar to the Wedenberg et al.^2^ and McNamara et al.^6^ models, our model predicts higher RBE‐weighted doses compared to RBE = 1.1 at the distal edges of each field, but our model's predictions were the greatest among them. For instance, the maximum RBE‐weighted doses to the genitalia (immediately distal to the treatment volume) predicted via our model was 150.7% of the prescribed dose, compared to 129.8% and 127.1% for the Wedenberg et al.^2^ and McNamara et al.^6^ models, respectively. However, these data serve only to demonstrate the feasibility of implementing our model within a clinical plan evaluation workflow, and future work must be done to establish if any differences between these models’ predictions might be clinically relevant.

## 4 DISCUSSION

Aside from the physical contribution of beam quality parameters such as LET to RBE, other biological factors are known to modulate cell radiosensitivity as well, including histologic type and genotype,^12^, ^13^, ^28^, ^29^ DNA repair capacity,^12^, ^13^, ^29^, ^30^, ^31^ anatomic site, tumorigenicity, and species of origin. Despite these biological factors driving cell radiosensitivity, we still observed strong linear correlations between radiosensitivity to protons and x‐rays even in the presence of great biological differences in these characteristics across cell lines. Notably, within the data we collected were cell lines with dramatically different DNA repair capacities, including the M059K and M059J cell lines which are nonhomologous end‐joining (NHEJ) repair proficient and deficient, respectively; and the HT1080‐shDNA‐PKcs, HT1080‐shRAD51^IND^, and HT1080 wild‐type cell lines which are deficient in NHEJ, homologous recombination, or proficient in both, respectively. Although one might expect these differences in DNA repair capacity to have a differential impact between radiation qualities since DNA repair capacity is known to greatly affect cell radiosensitivity and the way RBE depends on proton LET,^13^ remarkably, their presence does not seem to perturb the linear trends we observed (Figure 1). However, it is important to note that the absence (or presence) of DNA repair proteins affects cell radiosensitivity to both protons and photons. In a recent publication, we showed that the relative spread in cell radiosensitivity, even among cells with differing DNA‐repair status, is indistinguishable between photon, proton, and even much higher LET carbon ion radiation, and also that the relative importance of DNA repair capacity in determining cell radiosensitivity is not significantly diminished for higher LET carbon ions.^12^ In light of these findings, it may be expected that differential DNA‐repair capacity does not perturb the linear relationship between proton and x‐ray survival endpoints (e.g., D_10%, proton_ and D_10%, x‐ray_), as it implies that if differential DNA‐repair capacity renders cells differentially radiosensitive to x‐rays, it would do so by the same relative amount for protons, which would in turn preserve the proportionality of these endpoints.

That a simple proportionality relationship governs the RBE for a given dose‐weighted LET regardless of these biological differences implies that whatever the contribution from these biological factors to a cell's intrinsic radiosensitivity, when considering a cell's radiosensitivity to protons, the physical component dictated by the beam quality does not depend strongly on these other factors—the same proportionality relationship applies to all cell lines, independent of biology, at each dose‐weighted LET. Therefore, our work suggests a nuanced relationship between the biological factors governing a cell's intrinsic radiosensitivity and the physical factors governing the LET effect in determining a cell's radiosensitivity to protons: biological factors determine a cell's intrinsic radiosensitivity (e.g., D_10%, x‐ray_); physical factors determine generally how cell radiosensitivity depends on LET (e.g., the slope of D_10%,proton_ and D_10%,x‐ray_); but biological factors, in determining a particular cell's intrinsic radiosensitivity, largely dictate how that cell's radiosensitivity will vary with LET.

In line with this, one of the consequences of the trends underpinning our model is that they predict a non‐linear relationship between proton RBE and photon radiosensitivity, which follows from the linear relationship between radiosensitivity to protons and x‐rays. This can be seen by rearranging Equation (8) to describe how RBE depends on the slope and intercept functions and a cell's radiosensitivity to x‐rays, for example:

(11) $$\begin{array}{c} \mathrm{RB}E_{D10\%}=\frac{1}{c\cdot e^{-f\cdot \mathrm{LET}}+\frac{p\cdot \mathrm{LET}}{D_{10\%,x-\mathrm{rays}}}} \end{array}$$

This relationship predicts higher RBE values for radioresistant cells, lower RBE values for radiosensitive cells, and, to the best of our knowledge, unique among empirical models, it predicts sub‐unity RBE values for extraordinarily radiosensitive cells. Similar observations have been made in the context of heavier ions as well as protons, with near (and sometimes sub‐) unity RBE for radiosensitive cells deficient in DNA repair pathways^13^, ^29^, ^32^, ^33^, ^34^ and comparatively large RBE values for radioresistant cells.^13^, ^35^, ^36^, ^37^ Notably, here, while other empirical models suggest that the shape of the survival curve dictates its LET dependence (e.g., via α/β), ours suggests that a cell's intrinsic radiosensitivity (e.g., D_10%_) may also greatly influence that cell's LET dependence.

The major difference of our model relative to other empirical approaches stems from differences in the quantities whose LET dependence was characterized. Notably, rather than characterizing how the cell survival curve parameters, for example, the LQM parameters α and β, vary with LET, our formalism relies on characterizing how radiosensitivity metrics that are derived from them, for example, D_10%_, vary between radiation qualities. The uncertainty in estimating the LQM parameters from cell survival data is relatively large. Thus, characterizing the LET dependence of α and β suffers greatly from the noise in the experimental data. However, even though the estimation of radiosensitivity metrics such as D_10%_ from cell survival data rely on the estimation of the LQM parameters, the covariance of α and β as estimated from a cell survival curve tend to be strongly negative. Consequently, because of how these covariance terms are incorporated into the standard error propagation formula,^38^ the negative covariances between α and β lead to smaller overall uncertainties in estimating radiosensitivity metrics such as D_10%_. Thus, the relative uncertainty in estimating survival levels (e.g., D_10%_) is much less than the relative uncertainty in estimating α or β alone. This means that by modeling the LET dependence of radiosensitivity metrics such as D_10%_, as opposed to the LET dependence of the LQM parameters α and β, our approach is able to characterize underlying trends in data that are inherently less noisy than the trends accounted for by other empirical approaches, which greatly contributes to the improved accuracy of our approach.

A limitation of this work is that even though our model was trained over a large range of LET values (up to 37.8 keV/μm), the LET range over which we evaluated the linear correlations between proton and x‐ray radiosensitivity within a consistent experimental framework was limited (up to 9.9 keV/μm). This limitation was because very high LET values for protons can only be achieved at the end of their range, where very high dose and LET gradients make measuring survival difficult. Thus, to ensure the robustness of our dataset, we chose to limit our highest LET values to 9.9 keV/µm to minimize these dosimetric uncertainties arising from the experimental setup. A more thorough characterization of these trends at higher LET values might provide insight into the optimal choice of functions describing the LET dependence of the correlations’ slope and intercept. This may be of particular importance to the intercept's parameterization since the large noise within the literature data renders it challenging to convincingly determine which function best models how (or whether) the intercept of these linear correlations vary with LET.

An additional limitation of this work is with respect to inconsistencies in how LET is reported among literature data, in many cases with the dose‐weighted LET (LET_d_) not being reported at all. In Paganetti's review,^1^ for cases where the LET values were not reported, he approximated the irradiation conditions and performed Monte Carlo to estimate the LET_d_ values for the described radiation conditions.^1^ For the PIDE database, in such cases, the LET values were calculated using the stopping power code ATIMA.^39^ While these different approaches to calculate LET_d_ may contribute to the noise in the training dataset, they do not confound our conclusions with respect to the relative strengths of different models, as all models were retrained and tested against the same data.

There are several limitations with regards to the ultimate applicability of our model in a clinical setting. First, although our preliminary findings may imply that the RBE values in the distal edge of clinically realistic proton beams can be much greater than 1.1, a more detailed clinical study is needed to establish under what circumstances these differences may be sufficient to warrant the departure from RBE = 1.1 in a clinical context.

Second, our model requires photon survival data as an input parameter; in a clinical setting, this type of survival data is not available. Nevertheless, this limitation is not specific to our model, as most empirical models describe how cell survival varies from its photon baseline. In the case of our model, given that the linear relationship between proton and photon radiosensitivity is observed in cell lines of various histologic subtypes, anatomic sites, DNA repair capacities, and genotypes, we suspect that the universality of this phenomenon implies that this is likely the only input we need to characterize an individual cell line's biological response.

Finally, the extent to which our model can be used to predict tumor response in vivo is unknown. In vivo, additional factors contribute to a tumor's response to radiation, including tumor z factors,^40^ tumor oxygenation,^41^ and involvement of the immune system,^42^ none of which our model makes any attempt to account for. Because our model accounts for the variability in intrinsic radiosensitivity, which some suggest is the driving factor in dictating tumor response to radiation,^43^ we suspect that our model will largely be compatible with predicting the response of tumors via their intrinsic radiosensitivity response. Nevertheless, future work is still needed to clarify the extent to which our model can be translated in vivo.

As a final note, because the phenomenon underpinning our model were first observed by Suzuki et al.^19^ for carbon ions, we believe that our model can probably be extended to ions other than protons. If our model is to be extended to heavier ions, then future work is needed to determine to what extent dose‐weighted LET is sufficient as a beam quality specifier in the context of our model, specifically, whether we can reconcile the response to different ions by using an ion‐independent substitute for beam quality such as the Q factor proposed by Luhr et al.,^44^ or whether we might simply need to model the response to different ions separately.

## 5 CONCLUSION

Our findings show that radiosensitivity to protons is strongly correlated to radiosensitivity to x‐rays, and that this relationship can be used to model radiosensitivity, RBE and the survival curve of cells exposed to protons based on their response to photons. We showed that this model can be used to predict the proton response of cells, even those with vastly different intrinsic radiosensitivities or DNA repair deficiencies, within 15%–30%, and that our model is more accurate than previously established empirical proton RBE models. Our data may further suggest that biological factors, in addition to physical factors, have important roles in determining how cell radiosensitivity varies with LET.

## CONFLICT OF INTEREST

Gabriel O. Sawakuchi and Simona F. Shaitelman have received research funds from TAE Life Sciences, Artios Pharma and Alpha Tau Medical. Simona F. Shaitelman has received research funds from Varian Medical Systems, Inc.

## Supporting information

Supporting Information
