# Supplementary material for: An empirical model of proton RBE based on the linear correlation between x‐ray and proton radiosensitivity
Source: Med Phys. 2022 Jul 28;49(9):6221–36. doi: 10.1002/mp.15850 (PMC10360139; doi:10.1002/mp.15850)
Supplement: Supplementary file 1 — Supporting Information [file MP-49-6221-s001.pdf]

SUPPLEMENTAL INFORMATION

Supplemental Note 1: Details of training dataset

As described in our manuscript, the data included in this work's training dataset are a combination of data compiled in the PIDE database, Paganetti's Review, as well as other published work. For the data in the PIDE, the data were reanalyzed by the authors to assess the survival curve parameter values within a consistent framework. In this work, to ensure the data were as self-consistent as possible, we used those reanalyzed data as part of our training dataset. However, in cases where the reanalyzed β values were given as null or negative, we instead used the published values, since this would prevent the exclusion of the data by our criteria that $\beta_{x\text{-}ray} > 0$ and $\beta_{proton} \geq 0$ which ensures that the survival curves are integrable. These data are summarized below in Supplemental Table 1.

Supplemental Table 1: X-ray and proton cell survival curve parameters for data used in this work taken from the PIDE database v 3.2, Paganetti's 2014 review, and from other published work. The original publications are denoted in the same way they are listed in the PIDE.

Publication	Cell Line	X-ray Source	$\alpha_{x\text{-}ray}$ (Gy^{-1})	$\beta_{x\text{-}ray}$ (Gy^{-2})	Proton LET$_d$ (keV/μm)	α_{proton} (Gy^{-1})	β_{proton} (Gy^{-2})
goodhead92	HeLa	250kVp	5.35979E-01	2.77512E-02	20.270	5.15203E-01	8.59699E-02
goodhead92	HeLa	250kVp	5.35979E-01	2.77512E-02	23.915	6.16785E-01	6.30344E-02
goodhead92	C3H10T1/2	250kVp	1.75083E-01	3.28097E-02	22.090	4.97965E-01	0.00000E+00
folkard96	V79	240kVp	1.32917E-01	4.97218E-02	10.100	2.95146E-01	4.58630E-02
folkard96	V79	240kVp	1.32917E-01	4.97218E-02	17.800	4.61057E-01	2.57275E-02
folkard96	V79	240kVp	1.32917E-01	4.97218E-02	27.600	7.74597E-01	8.05804E-03
bettega98	C3H10T1/2	60Co	2.40000E-01	1.60000E-02	11.000	4.70000E-01	1.90000E-02
bettega98	C3H10T1/2	60Co	2.40000E-01	1.60000E-02	19.700	4.30000E-01	3.80000E-02
bettega98	C3H10T1/2	60Co	2.40000E-01	1.60000E-02	28.800	5.50000E-01	5.30000E-02
bettega98	C3H10T1/2	60Co	2.40000E-01	1.60000E-02	31.600	6.70000E-01	0.00000E+00
bettega98	C3H10T1/2	60Co	2.40000E-01	1.60000E-02	32.500	7.50000E-01	0.00000E+00
bettega98	C3H10T1/2	60Co	2.40000E-01	1.60000E-02	33.200	1.02000E+00	0.00000E+00
belli00	HF19	60Co	5.60939E-01	1.82066E-02	7.700	5.52877E-01	0.00000E+00
belli00	HF19	60Co	5.60939E-01	1.82066E-02	19.500	5.27624E-01	0.00000E+00
belli00	SCC25	60Co	6.46066E-01	1.95629E-02	7.700	5.29842E-01	6.13496E-02
belli00	SCC25	60Co	5.70000E-01	3.10000E-02	19.700	8.70000E-01	0.00000E+00
belli00	SCC25	60Co	6.46066E-01	1.95629E-02	29.500	8.01457E-01	0.00000E+00
belli00	SQ20B	60Co	1.00668E-01	2.36603E-02	7.700	1.61966E-01	8.50233E-03
belli00	SQ20B	60Co	1.00668E-01	2.36603E-02	19.800	2.58118E-01	0.00000E+00
belli00	SQ20B	60Co	1.30000E-01	1.70000E-02	30.000	5.70000E-01	0.00000E+00
belli98	V79	Not Specified	1.06097E-01	5.16814E-02	7.700	2.85536E-01	2.48850E-02
belli98	V79	Not Specified	1.06097E-01	5.16814E-02	11.000	4.38325E-01	2.24156E-02
belli98	V79	Not Specified	1.06097E-01	5.16814E-02	20.000	5.01822E-01	3.31919E-02
belli98	V79	Not Specified	1.06097E-01	5.16814E-02	30.500	7.32347E-01	0.00000E+00
belli98	V79	Not Specified	1.29000E-01	4.60000E-02	34.600	6.53000E-01	0.00000E+00
belli98	V79	Not Specified	1.29000E-01	4.60000E-02	37.800	5.80000E-01	0.00000E+00
perris86	V79	60Co	1.18132E-01	8.82831E-03	6.000	0.00000E+00	6.54872E-02
perris86	V79	60Co	1.18132E-01	8.82831E-03	12.000	4.08792E-01	0.00000E+00
wouters96	V79	60Co	1.32130E-01	2.11504E-02	2.330	6.37030E-02	4.23023E-02
wouters96	V79	60Co	1.32130E-01	2.11504E-02	2.730	6.73342E-02	4.25862E-02
wouters96	V79	60Co	1.32130E-01	2.11504E-02	2.890	6.63713E-02	4.26264E-02
wouters96	V79	60Co	1.32130E-01	2.11504E-02	3.090	5.83230E-02	4.43462E-02
wouters96	V79	60Co	1.32130E-01	2.11504E-02	3.290	3.66509E-02	4.75992E-02
wouters96	V79	60Co	1.32130E-01	2.11504E-02	3.440	4.61307E-02	4.68041E-02
wouters96	V79	60Co	1.32130E-01	2.11504E-02	3.700	4.05252E-02	4.72358E-02
wouters96	V79	60Co	1.32130E-01	2.11504E-02	4.000	5.22742E-02	4.68318E-02
wouters96	V79	60Co	1.32130E-01	2.11504E-02	4.410	6.53245E-02	4.56118E-02

Publication	Cell Line	X-ray Source	$\alpha_{x\text{-}ray}$ (Gy^{-1})	$\beta_{x\text{-}ray}$ (Gy^{-2})	Proton LET$_d$ (keV/µm)	α_{proton} (Gy^{-1})	β_{proton} (Gy^{-2})
wouters96	V79	60Co	1.32130E-01	2.11504E-02	5.110	7.08997E-02	4.62416E-02
wouters96	V79	60Co	1.32130E-01	2.11504E-02	6.230	1.03961E-01	4.42743E-02
miller95	C3H10T1/2	250kVp	8.64339E-02	6.86425E-02	15.000	8.36030E-01	0.00000E+00
prise90	V79	250kVp	1.10000E-01	2.70000E-02	16.900	3.50000E-01	4.50000E-02
prise90	V79	250kVp	1.10000E-01	2.70000E-02	23.700	3.30000E-01	6.60000E-02
prise90	V79	250kVp	1.10000E-01	2.70000E-02	31.000	1.03000E+00	0.00000E+00
ibanez09	B16-F0	137Cs	1.00000E-01	4.60000E-02	14.000	6.10000E-01	0.00000E+00
chaudhary14	AG01522	225kVp	6.63097E-01	8.04295E-02	1.110	7.49706E-01	1.16683E-01
chaudhary14	AG01522	225kVp	6.63097E-01	8.04295E-02	4.020	1.00825E+00	8.21381E-02
chaudhary14	AG01522	225kVp	6.63097E-01	8.04295E-02	7.000	1.36087E+00	0.00000E+00
chaudhary14	AG01522	225kVp	5.40000E-01	6.20000E-02	11.900	1.70000E+00	7.90000E-02
chaudhary14	AG01522	225kVp	6.63097E-01	8.04295E-02	18.000	1.87617E+00	0.00000E+00
chaudhary14	AG01522	225kVp	5.40000E-01	6.20000E-02	22.600	2.43000E+00	5.70000E-02
chaudhary14	AG01522	225kVp	6.63097E-01	8.04295E-02	1.200	6.45521E-01	1.17365E-01
chaudhary14	AG01522	225kVp	6.63097E-01	8.04295E-02	2.600	8.93760E-01	6.91900E-02
chaudhary14	AG01522	225kVp	6.63097E-01	8.04295E-02	4.500	1.17843E+00	3.92805E-02
chaudhary14	AG01522	225kVp	6.63097E-01	8.04295E-02	13.400	1.31731E+00	5.36229E-02
chaudhary14	AG01522	225kVp	6.63097E-01	8.04295E-02	21.700	1.65626E+00	0.00000E+00
chaudhary14	AG01522	225kVp	5.40000E-01	6.20000E-02	25.900	2.01000E+00	1.10000E-02
chaudhary14	U-87	225kVp	1.06353E-01	5.56575E-02	1.110	1.72995E-01	5.59509E-02
chaudhary14	U-87	225kVp	1.06353E-01	5.56575E-02	4.020	1.93583E-01	6.23440E-02
chaudhary14	U-87	225kVp	1.06353E-01	5.56575E-02	7.000	2.36111E-01	6.69091E-02
chaudhary14	U-87	225kVp	1.06353E-01	5.56575E-02	11.900	6.43904E-01	0.00000E+00
chaudhary14	U-87	225kVp	1.06353E-01	5.56575E-02	18.000	6.96989E-01	2.71646E-02
chaudhary14	U-87	225kVp	1.06353E-01	5.56575E-02	22.600	9.69226E-01	0.00000E+00
chaudhary14	U-87	225kVp	1.06353E-01	5.56575E-02	1.200	1.65379E-01	5.64698E-02
chaudhary14	U-87	225kVp	1.06353E-01	5.56575E-02	2.600	1.88606E-01	5.80320E-02
chaudhary14	U-87	225kVp	1.06353E-01	5.56575E-02	4.500	2.18782E-01	6.21542E-02
chaudhary14	U-87	225kVp	1.06353E-01	5.56575E-02	13.400	4.68959E-01	3.56728E-02
chaudhary14	U-87	225kVp	1.06353E-01	5.56575E-02	21.700	7.81564E-01	6.55704E-03
chaudhary14	U-87	225kVp	1.06353E-01	5.56575E-02	25.900	8.88187E-01	5.59466E-03
folkard89	V79	250kVp	1.10000E-01	2.70000E-02	17.000	1.30000E-01	7.80000E-02
folkard89	V79	250kVp	1.10000E-01	2.70000E-02	24.000	3.30000E-01	6.60000E-02
folkard89	V79	250kVp	1.10000E-01	2.70000E-02	32.000	1.03000E+00	0.00000E+00
gerelchuluun11	ONS76	10MV	1.27515E-01	3.90236E-02	2.200	1.05274E-01	4.98177E-02
gerelchuluun11	MOLT4	10MV	8.98081E-01	4.78125E-01	2.200	1.87025E+00	0.00000E+00
gerelchuluun15	V79	137Cs	1.13469E-01	2.68619E-02	2.200	1.20695E-01	2.91183E-02
gerelchuluun15	AA8	137Cs	3.38935E-01	2.25024E-02	2.200	2.09318E-01	3.30433E-02
gerelchuluun15	irs1SF	137Cs	9.08577E-01	6.28074E-02	2.200	7.46874E-01	9.93298E-02
gerelchuluun15	XR1	137Cs	1.04000E+00	3.40000E-04	2.200	1.01000E+00	0.00000E+00
gerelchuluun15	V3	137Cs	1.93000E+00	1.80000E-01	2.200	1.38000E+00	3.40000E-01
manti12	AG01522	225kVp	2.18234E-01	7.54073E-02	4.000	5.97088E-01	2.17085E-02
slonina14	HFIB2	6MV	6.65478E-01	3.72547E-02	7.900	1.09424E+00	0.00000E+00
slonina14	HFIB2	6MV	6.11727E-01	4.39981E-02	2.250	9.08110E-01	0.00000E+00
slonina14	HFIB2	6MV	6.11727E-01	4.39981E-02	2.930	9.74119E-01	0.00000E+00
slonina14	HFIB2	6MV	6.11727E-01	4.39981E-02	7.500	8.06720E-01	2.18679E-02
slonina14	HFIB15	6MV	5.95614E-01	5.35506E-02	7.900	6.62232E-01	5.79987E-02
slonina14	HFIB15	6MV	3.97084E-01	6.86470E-02	2.250	5.07915E-01	5.20648E-02
slonina14	HFIB15	6MV	3.97084E-01	6.86470E-02	2.930	5.07035E-01	5.31307E-02
slonina14	HFIB15	6MV	3.97084E-01	6.86470E-02	7.500	7.66285E-01	3.18880E-02
slonina14	HFIB30	6MV	8.31296E-01	4.39135E-02	7.900	1.15691E+00	0.00000E+00
slonina14	HFIB30	6MV	7.79551E-01	3.81740E-02	2.250	8.34182E-01	4.06865E-02
slonina14	HFIB30	6MV	7.79551E-01	3.81740E-02	2.930	8.60600E-01	3.26131E-02
slonina14	HFIB30	6MV	7.79551E-01	3.81740E-02	7.500	1.16439E+00	0.00000E+00
wouters15	V79	60Co	7.72841E-02	4.96975E-02	1.100	0.00000E+00	4.63857E-02
wouters15	V79	60Co	7.72841E-02	4.96975E-02	2.060	9.26454E-02	5.60108E-02
wouters15	V79	60Co	7.72841E-02	4.96975E-02	2.410	1.07685E-01	5.52736E-02
wouters15	V79	60Co	7.72841E-02	4.96975E-02	3.200	1.16314E-01	5.71015E-02
wouters15	V79	60Co	7.72841E-02	4.96975E-02	4.740	1.32776E-01	5.92868E-02
wouters15	V79	60Co	7.72841E-02	4.96975E-02	1.030	7.20624E-02	3.54752E-02
wouters15	V79	60Co	7.72841E-02	4.96975E-02	1.950	1.02835E-01	5.40978E-02
wouters15	V79	60Co	7.72841E-02	4.96975E-02	2.280	1.11447E-01	5.52001E-02
wouters15	V79	60Co	7.72841E-02	4.96975E-02	2.950	1.04681E-01	5.87724E-02
wouters15	V79	60Co	7.72841E-02	4.96975E-02	4.020	1.11819E-01	6.30537E-02
yashkin95	clone431	60Co	1.02322E-01	2.08173E-02	0.500	6.53201E-02	3.01520E-02
baggio02	DLD1	60Co	4.87388E-01	9.31984E-02	7.700	3.44371E-01	0.00000E+00
baggio02	HCT116	60Co	9.58349E-01	8.99989E-02	7.700	4.51848E-01	2.73438E-02
bird80	V79	250kVp	7.28171E-02	1.55686E-02	10.200	1.13808E-01	1.56560E-02
bird80	V79	250kVp	4.45049E-01	1.61343E-02	10.200	5.00510E-01	1.37553E-02

Publication	Cell Line	X-ray Source	$\alpha_{x\text{-}ray}$ (Gy^{-1})	$\beta_{x\text{-}ray}$ (Gy^{-2})	Proton LET_d (keV/µm)	α_{proton} (Gy^{-1})	β_{proton} (Gy^{-2})
doria12	V79	225kVp	9.64076E-02	3.49917E-02	10.000	0.00000E+00	2.32675E-01
doria12	V79	225kVp	9.64076E-02	3.49917E-02	14.000	3.48498E-01	0.00000E+00
doria12	V79	225kVp	9.64076E-02	3.49917E-02	20.000	4.32429E-01	0.00000E+00
doria12	V79	225kVp	9.64076E-02	3.49917E-02	28.000	2.50827E-01	4.67081E-02
hei88	HSF	137Cs	2.13526E-01	3.83856E-02	10.000	3.38221E-01	7.10327E-02
inada81	HMF	200kVp	3.23227E-01	4.02166E-02	1.300	1.69972E-01	6.19614E-02
inada81	HMF	200kVp	3.23227E-01	4.02166E-02	13.500	2.65476E-01	5.41779E-02
jeynes13	V79	300kVp	1.18021E-01	2.49424E-02	17.600	1.34435E-01	6.11482E-02
schuff02	V79	137Cs	1.50000E-01	3.00000E-02	3.440	1.40000E-01	4.50000E-02
guan15	H460	137Cs	4.40488E-01	5.41302E-02	0.900	2.21061E-01	1.13394E-01
guan15	H460	137Cs	4.40488E-01	5.41302E-02	1.200	1.96005E-01	1.19971E-01
guan15	H460	137Cs	4.40488E-01	5.41302E-02	1.600	2.81105E-01	1.00894E-01
guan15	H460	137Cs	4.40488E-01	5.41302E-02	1.800	2.01863E-01	1.18688E-01
guan15	H460	137Cs	4.40488E-01	5.41302E-02	1.900	2.32896E-01	1.14853E-01
guan15	H460	137Cs	4.40488E-01	5.41302E-02	2.300	2.84753E-01	9.77386E-02
guan15	H460	137Cs	4.40488E-01	5.41302E-02	3.000	3.61753E-01	7.69167E-02
guan15	H460	137Cs	4.40488E-01	5.41302E-02	5.100	1.50261E-01	1.48519E-01
guan15	H460	137Cs	4.40488E-01	5.41302E-02	10.800	4.96446E-01	1.01896E-01
guan15	H460	137Cs	4.40488E-01	5.41302E-02	15.200	5.50393E-01	2.90581E-01
guan15	H460	137Cs	4.40488E-01	5.41302E-02	17.700	7.82551E-01	5.50411E-01
guan15	H460	137Cs	4.40488E-01	5.41302E-02	19.000	1.40963E+00	5.33488E-01
guan15	H1437	137Cs	5.00000E-02	4.10000E-02	0.900	7.70000E-02	2.80000E-02
guan15	H1437	137Cs	5.00000E-02	4.10000E-02	1.200	1.36000E-01	2.00000E-02
guan15	H1437	137Cs	5.00000E-02	4.10000E-02	1.600	6.70000E-02	2.70000E-02
guan15	H1437	137Cs	5.00000E-02	4.10000E-02	1.800	5.90000E-02	3.80000E-02
guan15	H1437	137Cs	5.00000E-02	4.10000E-02	1.900	9.40000E-02	3.10000E-02
guan15	H1437	137Cs	5.00000E-02	4.10000E-02	2.300	9.60000E-02	3.20000E-02
guan15	H1437	137Cs	5.00000E-02	4.10000E-02	3.000	1.11000E-01	3.30000E-02
guan15	H1437	137Cs	5.00000E-02	4.10000E-02	5.100	3.40000E-02	5.20000E-02
guan15	H1437	137Cs	5.00000E-02	4.10000E-02	10.800	1.19000E-01	5.40000E-02
guan15	H1437	137Cs	5.00000E-02	4.10000E-02	15.200	1.80000E-01	9.50000E-02
guan15	H1437	137Cs	5.00000E-02	4.10000E-02	17.700	3.28000E-01	1.49000E-01
guan15	H1437	137Cs	5.00000E-02	4.10000E-02	19.000	3.60000E-01	2.72000E-01
OurData	HT1080	6MV	9.09600E-02	2.44800E-02	1.200	5.05600E-02	3.90200E-02
OurData	HT1080-shRAD51	6MV	5.43600E-01	5.49000E-03	1.200	4.46000E-01	2.46400E-02
OurData	HT1080-shDNA-PKcs	6MV	4.70200E-01	1.18100E-01	1.200	6.86800E-01	7.66900E-02
OurData	H460	6MV	4.61200E-02	8.98500E-02	1.200	2.42300E-02	9.95700E-02
OurData	H1299	6MV	8.87500E-02	2.40000E-02	1.200	1.31100E-01	2.38500E-02
OurData	M059K	6MV	3.14400E-01	2.06100E-02	1.200	2.67900E-01	2.35100E-02
OurData	M059J	6MV	1.36000E+00	2.31800E-01	1.200	8.40200E-01	3.91900E-01
OurData	BxPC3	6MV	3.07900E-01	1.54100E-02	1.200	3.75900E-01	1.07000E-02
OurData	HT1080	6MV	9.09600E-02	2.44800E-02	2.600	1.59200E-01	3.55200E-02
OurData	HT1080-shRAD51	6MV	5.43600E-01	5.49000E-03	2.600	4.15400E-01	4.77200E-02
OurData	HT1080-shDNA-PKcs	6MV	4.70200E-01	1.18100E-01	2.600	7.42400E-01	7.26200E-02
OurData	H460	6MV	4.61200E-02	8.98500E-02	2.600	1.67300E-01	8.51200E-02
OurData	H1299	6MV	8.87500E-02	2.40000E-02	2.600	7.80300E-02	3.37300E-02
OurData	M059K	6MV	3.14400E-01	2.06100E-02	2.600	3.30400E-01	2.41600E-02
OurData	M059J	6MV	1.36000E+00	2.31800E-01	2.600	1.58100E+00	1.27400E-12
OurData	BxPC3	6MV	3.07900E-01	1.54100E-02	2.600	3.24400E-01	1.15700E-02
OurData	HT1080	6MV	9.09600E-02	2.44800E-02	9.900	7.57200E-02	8.85100E-02
OurData	HT1080-shRAD51	6MV	5.43600E-01	5.49000E-03	9.900	5.45200E-01	5.25700E-02
OurData	HT1080-shDNA-PKcs	6MV	4.70200E-01	1.18100E-01	9.900	9.02100E-01	2.51400E-02
OurData	H460	6MV	4.61200E-02	8.98500E-02	9.900	1.45600E-01	1.50300E-01
OurData	H1299	6MV	8.87500E-02	2.40000E-02	9.900	1.02300E-01	5.58800E-02
OurData	M059K	6MV	3.14400E-01	2.06100E-02	9.900	3.60800E-01	5.72700E-02
OurData	M059J	6MV	1.36000E+00	2.31800E-01	9.900	1.68300E+00	7.20300E-13
OurData	BxPC3	6MV	3.07900E-01	1.54100E-02	9.900	5.33800E-01	1.09300E-02
Liu15	A549	250kVp	3.59000E-01	2.60000E-02	2.300	4.45000E-01	1.00000E-02
Liu15	ABC-1	250kVp	1.07000E-01	3.70000E-02	2.300	2.90000E-02	5.10000E-02
Liu15	Calu-6	250kVp	1.46000E-01	4.40000E-02	2.300	2.07000E-01	4.70000E-02
Liu15	HCC-44	250kVp	1.65000E-01	1.60000E-02	2.300	2.88000E-01	1.00000E-03
Liu15	HCC827	250kVp	2.96000E-01	4.50000E-02	2.300	2.57000E-01	5.30000E-02
Liu15	MDA-MB-436	250kVp	4.95000E-01	2.70000E-02	2.300	6.18000E-01	2.10000E-02
Liu15	H1299	250kVp	2.41000E-01	9.00000E-03	2.300	1.86000E-01	1.60000E-02
Liu15	H1563	250kVp	3.72000E-01	4.40000E-02	2.300	7.37000E-01	1.00000E-02
Liu15	H1703	250kVp	1.39000E-01	3.00000E-02	2.300	2.43000E-01	2.40000E-02

Publication	Cell Line	X-ray Source	$\alpha_{x\text{-}ray}$ (Gy^{-1})	$\beta_{x\text{-}ray}$ (Gy^{-2})	Proton LET$_d$ (keV/µm)	α_{proton} (Gy^{-1})	β_{proton} (Gy^{-2})
Liu15	H1792	250kVp	1.42000E-01	3.10000E-02	2.300	1.14000E-01	4.20000E-02
Liu15	H1869	250kVp	1.27000E-01	3.20000E-02	2.300	2.03000E-01	3.60000E-02
Liu15	H1915	250kVp	2.47000E-01	3.40000E-02	2.300	3.54000E-01	2.50000E-02
Liu15	H2126	250kVp	2.57000E-01	2.50000E-02	2.300	2.73000E-01	2.30000E-02
Liu15	H23	250kVp	1.50000E-02	6.80000E-01	2.300	2.40000E-02	6.59000E-01
Liu15	H460	250kVp	2.77000E-01	2.40000E-02	2.300	3.20000E-01	2.00000E-02
Liu15	H520	250kVp	4.43000E-01	2.50000E-02	2.300	4.05000E-01	3.70000E-02
Liu15	PC9	250kVp	2.67000E-01	3.20000E-02	2.300	3.08000E-01	2.80000E-02
Liu15	AG01522	250kVp	5.17000E-01	4.40000E-02	2.300	4.19000E-01	5.40000E-02
Liu15	PD20+D2	250kVp	4.32000E-01	3.60000E-02	2.300	3.58000E-01	4.50000E-02
Liu15	PD20-D2	250kVp	3.49000E-01	2.40000E-02	2.300	4.36000E-01	2.60000E-02
Liu15	PD220+A	250kVp	2.08000E-01	4.10000E-02	2.300	2.28000E-01	3.90000E-02
Liu15	PD220-A	250kVp	1.30000E-01	5.80000E-02	2.300	2.89000E-01	3.90000E-02
Liu15	H1915/vector	250kVp	2.71000E-01	2.80000E-02	2.300	3.82000E-01	1.80000E-02
Liu15	H1915/BRCA1-C3	250kVp	2.95000E-01	1.50000E-02	2.300	3.03000E-01	1.40000E-02
Liu15	Calu-6_scramble	250kVp	2.28000E-01	2.80000E-02	2.300	3.05000E-01	3.20000E-02
Liu15	Calu-6_FANCD2_siRNA	250kVp	2.13000E-01	3.30000E-02	2.300	3.52000E-01	3.00000E-02
Liu15	H460_FANCD2_siRNA	250kVp	3.94000E-01	7.00000E-03	2.300	6.02000E-01	4.00000E-03
Hall78	V79	60Co	2.20000E-02	2.20000E-02	2.050	4.20000E-02	3.00000E-02
Hall78	V79	60Co	2.20000E-02	2.20000E-02	1.110	3.80000E-02	2.90000E-02
Wouters14	V79-WNRE	60Co	7.20000E-02	5.00000E-02	1.110	1.02000E-01	5.20000E-02
Wouters14	V79-WNRE	60Co	7.20000E-02	5.00000E-02	1.190	1.05000E-01	5.00000E-02
Wouters14	V79-WNRE	60Co	7.20000E-02	5.00000E-02	1.450	8.70000E-02	5.60000E-02
Wouters14	V79-WNRE	60Co	7.20000E-02	5.00000E-02	1.770	7.20000E-02	5.90000E-02
Wouters14	V79-WNRE	60Co	7.20000E-02	5.00000E-02	1.940	1.20000E-01	5.20000E-02
Wouters14	V79-WNRE	60Co	7.20000E-02	5.00000E-02	2.090	1.08000E-01	5.40000E-02
Wouters14	V79-WNRE	60Co	7.20000E-02	5.00000E-02	2.280	1.04000E-01	5.50000E-02
Wouters14	V79-WNRE	60Co	7.20000E-02	5.00000E-02	2.530	1.22000E-01	5.40000E-02
Wouters14	V79-WNRE	60Co	7.20000E-02	5.00000E-02	2.900	1.13000E-01	5.60000E-02
Wouters14	V79-WNRE	60Co	7.20000E-02	5.00000E-02	3.460	1.13000E-01	5.90000E-02
Wouters14	V79-WNRE	60Co	7.20000E-02	5.00000E-02	4.820	1.41000E-01	5.90000E-02
Wouters14	V79-WNRE	60Co	7.20000E-02	5.00000E-02	11.000	5.33000E-01	5.00000E-02
Wouters14	V79-WNRE	60Co	7.20000E-02	5.00000E-02	1.080	9.80000E-02	5.20000E-02
Wouters14	V79-WNRE	60Co	7.20000E-02	5.00000E-02	1.050	1.03000E-01	4.90000E-02
Wouters14	V79-WNRE	60Co	7.20000E-02	5.00000E-02	1.110	9.10000E-02	5.20000E-02
Wouters14	V79-WNRE	60Co	7.20000E-02	5.00000E-02	1.130	8.60000E-02	5.00000E-02
Wouters14	V79-WNRE	60Co	7.20000E-02	5.00000E-02	1.410	9.90000E-02	5.20000E-02
Wouters14	V79-WNRE	60Co	7.20000E-02	5.00000E-02	1.800	8.90000E-02	5.40000E-02
Wouters14	V79-WNRE	60Co	7.20000E-02	5.00000E-02	1.910	9.40000E-02	5.50000E-02
Wouters14	V79-WNRE	60Co	7.20000E-02	5.00000E-02	2.030	1.10000E-01	5.40000E-02
Wouters14	V79-WNRE	60Co	7.20000E-02	5.00000E-02	2.170	1.03000E-01	5.50000E-02
Wouters14	V79-WNRE	60Co	7.20000E-02	5.00000E-02	2.360	1.06000E-01	5.70000E-02
Wouters14	V79-WNRE	60Co	7.20000E-02	5.00000E-02	2.640	8.60000E-02	6.20000E-02
Wouters14	V79-WNRE	60Co	7.20000E-02	5.00000E-02	2.990	1.41000E-01	5.40000E-02
Wouters14	V79-WNRE	60Co	7.20000E-02	5.00000E-02	3.480	9.90000E-02	6.40000E-02
Wouters14	V79-WNRE	60Co	7.20000E-02	5.00000E-02	4.040	1.21000E-01	6.30000E-02
Wouters14	V79-WNRE	60Co	7.20000E-02	5.00000E-02	10.500	2.94000E-01	2.73000E-01
Grosse14	CHO	200kVp	5.90000E-02	3.40000E-02	2.530	1.52000E-01	3.40000E-02
Grosse14	CHO	200kVp	9.00000E-02	2.20000E-02	2.530	8.50000E-02	4.00000E-02
Grosse14	CHO	200kVp	1.01000E-01	2.20000E-02	2.530	1.42000E-01	3.20000E-02
Grosse14	CHO	200kVp	1.90000E-01	1.40000E-02	2.530	1.72000E-01	3.90000E-02
Grosse14	CHO-def1	200kVp	3.79000E-01	3.50000E-02	2.530	7.20000E-01	3.00000E-02
Grosse14	CHO-def2	200kVp	2.89000E-01	1.80000E-02	2.530	3.45000E-01	3.50000E-02
Grosse14	CHO-def3	200kVp	5.41000E-01	2.90000E-02	2.530	8.75000E-01	2.30000E-02
Grosse14	CHO-def4	200kVp	1.25800E+00	1.40000E-02	2.530	1.70800E+00	5.00000E-03
Grosse14	CHO-def5	200kVp	9.19000E-01	8.00000E-03	2.530	9.90000E-01	1.70000E-02
Grosse14	AA8-trans	200kVp	8.60000E-02	2.90000E-02	2.530	1.15000E-01	4.90000E-02
Gueulette96	CHO	60Co	1.32000E-01	7.20000E-02	1.290	1.03000E-01	9.50000E-02
Gueulette96	CHO	60Co	1.32000E-01	7.20000E-02	4.100	1.92000E-01	7.70000E-02
Gueulette96	CHO	60Co	1.32000E-01	7.20000E-02	2.730	7.10000E-02	8.50000E-02
Gueulette96	CHO	60Co	1.32000E-01	7.20000E-02	4.530	1.98000E-01	7.30000E-02
Coutrakon97	V79	60Co	5.10000E-02	2.50000E-02	1.090	1.28000E-01	3.30000E-02
Coutrakon97	V79	60Co	5.10000E-02	2.50000E-02	0.990	1.15000E-01	3.50000E-02
Coutrakon97	V79	60Co	5.10000E-02	2.50000E-02	1.130	1.20000E-01	3.50000E-02
Coutrakon97	V79	60Co	5.10000E-02	2.50000E-02	1.020	1.36000E-01	3.30000E-02
Coutrakon97	V79	60Co	5.10000E-02	2.50000E-02	1.140	1.38000E-01	3.10000E-02
Coutrakon97	V79	60Co	5.10000E-02	2.50000E-02	1.030	1.37000E-01	2.90000E-02

Publication	Cell Line	X-ray Source	$\alpha_{x\text{-}ray}$ (Gy^{-1})	$\beta_{x\text{-}ray}$ (Gy^{-2})	Proton LET$_d$ (keV/µm)	α_{proton} (Gy^{-1})	β_{proton} (Gy^{-2})
Coutrakon97	V79	60Co	5.10000E-02	2.50000E-02	1.120	1.25000E-01	3.10000E-02
Coutrakon97	V79	60Co	5.10000E-02	2.50000E-02	1.160	1.29000E-01	3.00000E-02
Coutrakon97	V79	60Co	5.10000E-02	2.50000E-02	1.750	1.54000E-01	2.70000E-02
Coutrakon97	V79	60Co	5.10000E-02	2.50000E-02	2.100	1.35000E-01	3.00000E-02
Coutrakon97	V79	60Co	5.10000E-02	2.50000E-02	2.710	1.13000E-01	3.20000E-02
Coutrakon97	V79	60Co	5.10000E-02	2.50000E-02	7.060	1.45000E-01	3.00000E-02
Coutrakon97	V79	60Co	5.10000E-02	2.50000E-02	0.990	1.03000E-01	3.30000E-02
Coutrakon97	V79	60Co	5.10000E-02	2.50000E-02	1.230	1.26000E-01	2.90000E-02
Coutrakon97	V79	60Co	5.10000E-02	2.50000E-02	1.110	1.08000E-01	3.20000E-02
Coutrakon97	V79	60Co	5.10000E-02	2.50000E-02	1.280	1.13000E-01	3.00000E-02
Coutrakon97	V79	60Co	5.10000E-02	2.50000E-02	1.700	1.19000E-01	3.10000E-02
Coutrakon97	V79	60Co	5.10000E-02	2.50000E-02	2.080	1.33000E-01	3.00000E-02
Coutrakon97	V79	60Co	5.10000E-02	2.50000E-02	2.630	1.20000E-01	3.20000E-02
Coutrakon97	V79	60Co	5.10000E-02	2.50000E-02	5.820	1.67000E-01	2.70000E-02
Coutrakon97	V79	60Co	5.10000E-02	2.50000E-02	1.160	1.28000E-01	2.70000E-02
Coutrakon97	V79	60Co	5.10000E-02	2.50000E-02	1.200	1.18000E-01	2.70000E-02
Coutrakon97	V79	60Co	5.10000E-02	2.50000E-02	1.790	1.29000E-01	2.60000E-02
Coutrakon97	V79	60Co	5.10000E-02	2.50000E-02	2.320	1.32000E-01	2.50000E-02
Coutrakon97	V79	60Co	5.10000E-02	2.50000E-02	3.200	1.69000E-01	2.50000E-02
Raju78	V79	60Co	1.06000E-01	4.00000E-02	1.180	2.13000E-01	4.00000E-02
Raju78	V79	60Co	1.06000E-01	4.00000E-02	2.120	9.10000E-02	4.90000E-02
Raju78	V79	250kVp	1.80000E-01	2.10000E-02	1.180	1.20000E-01	2.80000E-02
Raju78	V79	250kVp	1.80000E-01	2.10000E-02	2.120	6.50000E-02	3.30000E-02
Schettino01	V79	240kVp	1.30000E-01	4.80000E-02	25.400	7.33000E-01	0.00000E+00
Schettino01	V79	240kVp	1.30000E-01	4.80000E-02	10.900	2.41000E-01	4.60000E-02
belli89	V79-753B	200kVp	1.28000E-01	4.60000E-02	30.500	7.44000E-01	0.00000E+00
belli89	V79-753B	200kVp	1.28000E-01	4.60000E-02	20.000	4.71000E-01	4.40000E-02
belli89	V79-753B	200kVp	1.28000E-01	4.60000E-02	10.900	3.72000E-01	3.60000E-02
Ogheri97	V79	200kVp	1.28000E-01	4.60000E-02	31.000	7.44000E-01	0.00000E+00
goodhead92	V79-4	200kVp	1.29000E-01	4.60000E-02	22.910	3.00000E-01	5.20000E-02
goodhead92	V79-4	200kVp	1.29000E-01	4.60000E-02	20.270	4.20000E-01	1.90000E-02
yashkin95	clone431	60Co	7.80000E-02	2.40000E-02	5.220	1.00000E-01	2.50000E-02
yashkin95	clone431	60Co	7.80000E-02	2.40000E-02	1.090	7.10000E-02	3.00000E-02
matsumura99	V79	137Cs	9.60000E-02	2.80000E-02	11.000	2.04000E-01	2.10000E-02
matsumura99	V79	137Cs	9.60000E-02	2.80000E-02	4.250	2.26000E-01	2.00000E-02
matsumura99	V79	137Cs	9.60000E-02	2.80000E-02	1.250	1.51000E-01	2.50000E-02
Wainson72	B11	137CS	1.44000E-01	3.70000E-02	0.790	1.09000E-01	1.60000E-02
Wainson72	B11	137CS	1.44000E-01	3.70000E-02	3.100	2.43000E-01	1.30000E-02
Wainson72	Hela	137CS	3.23000E-01	4.50000E-02	0.790	1.88000E-01	3.80000E-02
Wainson72	Hela	137CS	3.23000E-01	4.50000E-02	0.790	1.88000E-01	3.80000E-02
Robertson94	V79	60Co	8.70000E-02	2.00000E-02	1.220	6.20000E-02	2.30000E-02
Robertson94	V79	60Co	8.70000E-02	2.00000E-02	1.120	7.10000E-02	2.50000E-02
Robertson94	V79	60Co	8.70000E-02	2.00000E-02	1.910	5.30000E-02	3.70000E-02
Robertson94	V79	60Co	8.70000E-02	2.00000E-02	2.410	6.20000E-02	4.00000E-02
Robertson94	V79	60Co	8.70000E-02	2.00000E-02	3.830	4.40000E-02	4.70000E-02
Robertson94	V79	60Co	8.70000E-02	2.00000E-02	1.170	9.00000E-02	1.90000E-02
Robertson94	V79	60Co	8.70000E-02	2.00000E-02	1.120	9.60000E-02	1.90000E-02
Robertson94	V79	60Co	8.70000E-02	2.00000E-02	1.070	6.80000E-02	2.30000E-02
Robertson94	V79	60Co	8.70000E-02	2.00000E-02	1.190	8.30000E-02	2.40000E-02
Robertson94	V79	60Co	8.70000E-02	2.00000E-02	1.690	7.90000E-02	3.30000E-02
Robertson94	V79	60Co	8.70000E-02	2.00000E-02	2.060	8.10000E-02	4.00000E-02
Robertson94	V79	60Co	8.70000E-02	2.00000E-02	2.540	5.00000E-02	4.60000E-02
Robertson94	V79	60Co	8.70000E-02	2.00000E-02	6.080	6.00000E-02	4.80000E-02
Robertson94	V79	60Co	8.70000E-02	2.00000E-02	1.070	9.90000E-02	1.90000E-02
Robertson94	V79	60Co	8.70000E-02	2.00000E-02	1.080	9.90000E-02	2.00000E-02
Robertson94	V79	60Co	8.70000E-02	2.00000E-02	0.990	7.30000E-02	2.20000E-02
Robertson94	V79	60Co	8.70000E-02	2.00000E-02	1.080	8.70000E-02	2.00000E-02
Robertson94	V79	60Co	8.70000E-02	2.00000E-02	1.050	7.00000E-02	2.10000E-02
Robertson94	V79	60Co	8.70000E-02	2.00000E-02	1.090	4.70000E-02	2.40000E-02
Robertson94	V79	60Co	8.70000E-02	2.00000E-02	1.160	4.00000E-02	2.40000E-02
Robertson94	V79	60Co	8.70000E-02	2.00000E-02	1.130	3.70000E-02	2.50000E-02
Robertson94	V79	60Co	8.70000E-02	2.00000E-02	1.720	4.20000E-02	2.80000E-02
Robertson94	V79	60Co	8.70000E-02	2.00000E-02	1.880	2.70000E-02	3.90000E-02
Robertson94	V79	60Co	8.70000E-02	2.00000E-02	2.760	1.90000E-02	4.30000E-02
Robertson94	V79	60Co	8.70000E-02	2.00000E-02	7.750	4.00000E-02	3.70000E-02
Tang97	CHO	137Cs	1.60000E-01	2.50000E-02	1.740	1.99000E-01	2.20000E-02
Tang97	CHO	137Cs	1.60000E-01	2.50000E-02	3.110	2.07000E-01	2.50000E-02
Tang97	CHO	137Cs	1.60000E-01	2.50000E-02	3.790	2.17000E-01	2.60000E-02
Tang97	CHO	137Cs	1.60000E-01	2.50000E-02	4.760	2.49000E-01	2.50000E-02

Publication	Cell Line	X-ray Source	$\alpha_{x\text{-}ray}$ (Gy^{-1})	$\beta_{x\text{-}ray}$ (Gy^{-2})	Proton LET$_d$ (keV/µm)	α_{proton} (Gy^{-1})	β_{proton} (Gy^{-2})
Sgura00	C1-1	60Co	2.30000E-01	3.00000E-02	27.600	5.39000E-01	0.00000E+00
Sgura00	C1-1	60Co	2.30000E-01	3.00000E-02	7.700	1.94000E-01	2.30000E-02
ando01	V79	6MV	2.10000E-01	1.60000E-02	2.020	2.14000E-01	2.10000E-02
ando01	V79	6MV	2.10000E-01	1.60000E-02	1.020	1.66000E-01	2.40000E-02
ando01	SCC61	6MV	1.62000E-01	1.11000E-01	2.020	4.73000E-01	8.50000E-02
ando01	SCC61	6MV	1.62000E-01	1.11000E-01	1.020	2.45000E-01	9.70000E-02
ando01	NB1RGB	6MV	3.52000E-01	3.50000E-02	2.020	1.00000E+00	4.10000E-02
ando01	NB1RGB	6MV	3.52000E-01	3.50000E-02	1.020	4.01000E-01	4.00000E-02
perris86	V79	60Co	1.40000E-01	6.00000E-03	12.100	4.30000E-01	0.00000E+00
perris86	V79	60Co	1.40000E-01	6.00000E-03	5.800	2.10000E-01	2.30000E-02
hei88	C3H10T1/2	250kVp	1.80000E-01	4.10000E-02	10.000	3.10000E-01	3.00000E-02
robertson75	H4	60Co	1.85000E-01	2.60000E-02	1.210	1.70000E-02	5.30000E-02
robertson75	H4	60Co	1.85000E-01	2.60000E-02	1.450	0.00000E+00	4.00000E-02
robertson75	H4	60Co	1.85000E-01	2.60000E-02	2.280	7.10000E-02	3.90000E-02
robertson75	H4	60Co	1.85000E-01	2.60000E-02	3.050	1.39000E-01	4.40000E-02
robertson75	H4	60Co	1.85000E-01	2.60000E-02	3.510	0.00000E+00	5.40000E-02
urano80	C3HF	60Co	1.51000E-01	1.90000E-02	2.200	2.12000E-01	2.50000E-02
urano80	C3HF	60Co	1.84000E-01	1.60000E-02	2.200	2.11000E-01	1.70000E-02
urano80	C3HF	60Co	1.84000E-01	1.60000E-02	1.190	2.03000E-01	2.10000E-02
urano80	C3HF	60Co	2.68000E-01	1.40000E-02	2.200	2.82000E-01	2.10000E-02
bettega90	C3H10T1/2	60Co	2.40000E-01	1.60000E-02	1.830	5.00000E-02	4.10000E-02
satoh86	HMV	60Co	4.00000E-02	1.70000E-02	3.940	5.40000E-02	1.70000E-02
aoki-nakano14	HSG	4MV	5.20000E-02	9.00000E-02	2.560	3.40000E-02	9.80000E-02
aoki-nakano14	HSG	4MV	6.20000E-02	8.30000E-02	2.600	2.15000E-01	5.80000E-02
aoki-nakano14	HSG	4MV	8.30000E-02	8.40000E-02	2.560	0.00000E+00	1.14000E-01
aoki-nakano14	HSG	4MV	2.14000E-01	4.60000E-02	2.500	3.15000E-01	3.80000E-02
calugaru11	SQ20B	225 kVp	2.00000E-02	1.10000E-02	2.420	3.60000E-02	1.00000E-02
calugaru11	SQ20B	137Cs	2.00000E-02	1.10000E-02	4.040	2.90000E-02	1.20000E-02
calugaru11	SQ20B	137Cs	2.00000E-02	1.10000E-02	6.850	2.70000E-02	1.60000E-02
calugaru11	SQ20B	137Cs	2.00000E-02	1.10000E-02	1.140	4.40000E-02	9.00000E-03
calugaru11	SQ20B	137Cs	2.00000E-02	1.10000E-02	2.740	4.40000E-02	9.00000E-03
calugaru11	SQ20B	137Cs	2.00000E-02	1.10000E-02	5.800	4.40000E-02	9.00000E-03
calugaru11	HeLa	137Cs	2.28000E-01	3.30000E-02	2.420	2.53000E-01	2.40000E-02
calugaru11	HeLa	137Cs	2.28000E-01	3.30000E-02	4.040	2.93000E-01	2.00000E-02
calugaru11	HeLa	137Cs	2.28000E-01	3.30000E-02	6.850	3.52000E-01	2.10000E-02
calugaru11	HeLa	137Cs	2.28000E-01	3.30000E-02	1.140	2.13000E-01	5.40000E-02
calugaru11	HeLa	137Cs	2.28000E-01	3.30000E-02	2.740	2.13000E-01	5.40000E-02
calugaru11	HeLa	137Cs	2.28000E-01	3.30000E-02	5.800	2.13000E-01	5.40000E-02
coudi94	CAL4	60Co	1.18000E-01	4.70000E-02	1.300	1.04000E-01	4.20000E-02
coudi94	CAL4	60Co	1.18000E-01	4.70000E-02	2.300	5.70000E-02	5.70000E-02
coudi94	CAL4	60Co	1.18000E-01	4.70000E-02	2.650	4.80000E-02	6.20000E-02
coudi94	CAL4	60Co	1.18000E-01	4.70000E-02	3.750	3.50000E-02	7.20000E-02
coudi94	CAL4	60Co	1.18000E-01	4.70000E-02	6.000	9.60000E-02	7.40000E-02
zhang13	A549/H460	60Co	2.11000E-01	7.10000E-02	2.500	3.85000E-01	8.00000E-02
zhang13	A549/H460	60Co	2.95000E-01	1.80000E-02	2.500	3.89000E-01	2.70000E-02
inada81	HMV	200kVp	2.17000E-01	5.60000E-02	13.500	1.58000E-01	7.70000E-02
inada81	HMV	200kVp	2.17000E-01	5.60000E-02	1.300	4.80000E-02	8.80000E-02
kagawa02	HSG	4MV	1.89000E-01	4.70000E-02	1.110	1.68000E-01	5.30000E-02
kagawa02	HSG	4MV	1.89000E-01	4.70000E-02	2.110	1.80000E-01	5.10000E-02
kagawa02	HSG	4MV	1.89000E-01	4.70000E-02	2.540	1.76000E-01	5.20000E-02
kagawa02	HSG	4MV	1.89000E-01	4.70000E-02	3.910	1.55000E-01	6.00000E-02
kagawa02	HSG	4MV	1.89000E-01	4.70000E-02	4.800	1.70000E-01	5.70000E-02
kase13	HSG	200kVp	1.96000E-01	4.40000E-02	1.200	1.57000E-01	4.80000E-02
kase13	HSG	200kVp	1.96000E-01	4.40000E-02	2.030	1.64000E-01	4.80000E-02
kase13	HSG	200kVp	1.96000E-01	4.40000E-02	2.650	1.39000E-01	5.60000E-02
kase13	HSG	200kVp	1.96000E-01	4.40000E-02	4.250	1.41000E-01	6.20000E-02
matsura10	HSG	200kVp	1.96000E-01	4.40000E-02	0.560	2.37000E-01	4.90000E-02
matsura10	HSG	200kVp	1.96000E-01	4.40000E-02	3.190	3.48000E-01	4.70000E-02
matsura10	HSG	200kVp	1.96000E-01	4.40000E-02	0.560	1.83000E-01	5.30000E-02
matsura10	HSG	200kVp	1.96000E-01	4.40000E-02	3.190	2.94000E-01	5.60000E-02
baek08	HSG	6MV	2.36000E-01	4.70000E-02	1.140	2.73000E-01	4.60000E-02
baek08	HSG	6MV	2.36000E-01	4.70000E-02	2.560	2.91000E-01	4.10000E-02
butterworth12	DU-145	6MV	1.29000E-01	2.20000E-02	2.860	8.60000E-02	3.30000E-02
butterworth12	DU-145	6MV	1.29000E-01	2.20000E-02	2.860	1.47000E-01	2.10000E-02
matsumoto14	HSG	6MV	1.90000E-01	3.00000E-02	2.760	2.20000E-01	5.00000E-02
matsumoto14	HSG	6MV	1.90000E-01	3.00000E-02	3.270	2.80000E-01	5.00000E-02
matsumoto14	HSG	6MV	1.90000E-01	3.00000E-02	3.615	2.50000E-01	5.00000E-02
matsumoto14	HSG	6MV	1.90000E-01	3.00000E-02	4.070	2.60000E-01	5.00000E-02
matsumoto14	HSG	6MV	1.90000E-01	3.00000E-02	4.930	4.20000E-01	5.00000E-02

Publication	Cell Line	X-ray Source	$\alpha_{x\text{-}ray}$ (Gy^{-1})	$\beta_{x\text{-}ray}$ (Gy^{-2})	Proton LET_d (keV/μm)	α_{proton} (Gy^{-1})	β_{proton} (Gy^{-2})
matsumoto14	HSG	6MV	1.90000E-01	3.00000E-02	6.190	4.10000E-01	5.00000E-02
matsumoto14	HSG	6MV	1.90000E-01	3.00000E-02	7.900	3.80000E-01	5.00000E-02
matsumoto14	HSG	6MV	1.90000E-01	3.00000E-02	9.445	4.40000E-01	3.00000E-02
matsumoto14	HSG	6MV	1.90000E-01	3.00000E-02	10.800	4.20000E-01	3.00000E-02
ogata05	HT1080	4MV	2.26000E-01	3.00000E-02	2.560	2.18000E-01	3.90000E-02
yogo11	HSG	4MV	2.44000E-01	2.20000E-02	17.100	2.43000E-01	4.10000E-02
bettega00	SCC25-prog	60Co	1.70000E-01	1.50000E-02	1.930	1.10000E-01	2.40000E-02
bettega00	SCC25-prog	60Co	1.70000E-01	1.50000E-02	2.420	1.90000E-01	2.60000E-02
bettega00	SCC25-prog	60Co	1.70000E-01	1.50000E-02	6.710	1.40000E-01	3.90000E-02
bettega00	SCC25-prog	60Co	1.70000E-01	1.50000E-02	8.110	1.50000E-01	3.20000E-02
bettega00	SCC25-prog	60Co	1.70000E-01	1.50000E-02	8.830	3.40000E-01	1.50000E-01
bettega00	SCC25	60Co	5.70000E-01	1.20000E-02	1.930	5.70000E-01	1.20000E-02
bettega00	SCC25	60Co	5.70000E-01	1.20000E-02	2.420	6.10000E-01	1.00000E-02
bettega00	SCC25	60Co	5.70000E-01	1.20000E-02	6.710	7.00000E-01	1.80000E-02
bettega00	SCC25	60Co	5.70000E-01	1.20000E-02	8.110	8.30000E-01	1.00000E-03
bettega00	SCC25	60Co	5.70000E-01	1.20000E-02	8.830	1.23000E+00	0.00000E+00
Wera13	A549/H460	250kVp	3.32000E-01	1.80000E-02	10.000	8.24000E-01	0.00000E+00
Wera11	A549/H460	250kVp	3.32000E-01	1.80000E-02	25.500	1.26000E+00	0.00000E+00
yang07	AG01522	250kVp	2.55000E-01	6.30000E-02	0.200	5.20000E-01	2.90000E-02
Fuhrman	EUE	60Co	4.27000E-01	1.50000E-02	1.850	3.64000E-01	2.90000E-02

Supplemental Note 2: Details of fitting

All of the model fitting was performed in MATLAB 2020, using the functions *lsqnonlin*, *fminunc, fmincon*, *nlinfit and lsqcurvefit*. We used lsqnonlin, fminunc and fmincon to determine the free parameter values when minimizing the L2 norm of the data since these functions allow for objective functions to be implemented with more sophistication than simply the sum-of-square distance between the data and the predictions. *nlinfit* and lsqcurvefit were used when optimizing the models to predict the RBE at the 2 Gy dose level when the residual sum-of-squares was to be minimized.

As our model (as well as the Mairani model) contain a large number of free parameters, the process of fitting such multivariate functions to our noisy training data often depended strongly on the initial conditions selected, since it is possible for the fitting algorithms to converge on local minimums within the parameter space rather than the global minimum. To work around this potential issue, for each fit, we performed 10000 iterations of each fit, using the previously-fit model parameters as initial conditions, but with a randomly-generated amount of noise added to them (±10% of the initial conditions). This allowed for the fitting to be performed more broadly across the parameter space in an attempt to converge to the true global minimum distance between the predictions and the measured data. The set of model parameters resulting in the smallest L2 norm were then selected as the final parameter values.

Supplemental Note 3: Choice of functions to describe the LET dependence of the slope and intercept of the linear correlations between proton and x-ray radiosensitivity

When selecting functions to model for the slope and intercept's LET dependence, we sought functions using a minimum number of free number of parameters to produce the trends we observed and subject to a limited number of constraints. For the slope, we wished the function that described its LET dependence to tend towards non-negative values at high LET values. This is because a negative slope between, for example $D_{10\%,ion}$ and $D_{10\%,x\text{-}ray}$ implies that modulating a cell's radiosensitivity, for example via DNA-repair inhibition, would render cells more sensitive to x-rays but more resistant to protons which is nonsensical. We also wished that the slope would tend to positive values for LET values approaching zero for the same reason. For the intercept, we subjected it to the constraint that the intercept must be non-negative for all LET values. This is because for cells that are arbitrarily radiosensitive to x-rays, a negative intercept would imply that they have negative radiosensitivities to protons, which is non-sensical. These candidate functions are summarized in Table 1.

For every possible combination of slope and intercept function, we fit the set of free parameters to the training dataset's $D_{5\%}$, $D_{10\%}$, $D_{20\%}$, $D_{37\%}$ and $D_{50\%}$ values, and SF_{2Gy} values using Matlab's lsqnonlin function, minimizing the relative distance between the predicted endpoint and the measured endpoint. We calculated the Bayesian Information Criteria[24] associated with each endpoint's fit to determine which parameterizations best reproduced the underlying trends in the data. Table 2 and Supplemental Table 2 - Supplemental Table 6 show these values. Generally, the simple exponential function was the best function describing the slope's LET dependence (smallest BIC values), but from these analyses alone it is not clear whether the intercept is best modeled by a constant, a linear function or an exponential function. Further comparisons combining multiple endpoints into a single predictive model suggest that the linear function with no offset offers a suitable description of the data when predicting α and β using multiple endpoints (Supplemental Note 5).

Supplemental Table 2: Bayesian Information Criteria (BIC) values determined by fitting the function create by combining the candidate slope and intercept functions to the training dataset's $D_{5\%}$ values. Each cell corresponds to the BIC value associated with a given slope and intercept function combination. Green cells indicate smaller BIC values (better performing functions) while red cells indicate larger BIC values (poorer performing functions).

Bayesian Information Criterion (BIC) value associated with the function predicting $D_{10\%}$							
Intercept / Slope	m	$p \cdot LET$	$m + p \cdot LET$	$m + p \cdot LET + q \cdot LET^2$	$q\, e^{s \cdot LET}$	$q\, e^{s \cdot LET} + m$	$q \cdot e^{s \cdot LET} + p \cdot LET + m$
$c\, e^{-f \cdot LET}$	-151.3	-141.0	-148.0	-142.0	-147.1	-141.2	-136.0
$c\, e^{-f \cdot LET} + g$	-145.4	-135.0	-142.0	-136.0	-141.2	-135.2	-130.1
$c \cdot e^{-f \cdot LET - h \cdot LET^2} + g$	-139.7	-136.9	-137.2	-131.2	-136.7	-132.2	-125.3
$(c + h \cdot LET) \cdot e^{-f \cdot LET} + g$	-139.5	-135.2	-137.0	-131.0	-136.2	-132.0	-125.0
$c \cdot ln(LET - h) \cdot e^{-f \cdot LET} + g$	-139.5	-134.0	-136.8	-130.8	-135.9	-131.8	-124.8
$\frac{c}{\Gamma(f \cdot LET + h + 1)} + g$	-139.6	-136.3	-137.1	-131.2	-136.5	-132.1	-125.2
$\frac{c\, \lambda^{f \cdot (LET - h)} \cdot e^{-\lambda}}{\Gamma(f \cdot (LET - h) + 1)} + g$	-133.7	-130.9	-131.2	-125.2	-39.7	-126.2	-121.9

Supplemental Table 3: Bayesian Information Criteria (BIC) values determined by fitting the function create by combining the candidate slope and intercept functions to the training dataset's $D_{20\%}$ values. Each cell corresponds to the BIC value associated with a given slope and intercept function combination. Green cells indicate smaller BIC values (better performing functions) while red cells indicate larger BIC values (poorer performing functions).

Bayesian Information Criterion (BIC) value associated with the function predicting $D_{10\%}$							
Intercept / Slope	m	$p \cdot LET$	$m + p \cdot LET$	$m + p \cdot LET + q \cdot LET^2$	$q\, e^{s \cdot LET}$	$q\, e^{s \cdot LET} + m$	$q \cdot e^{s \cdot LET} + p \cdot LET + m$
$c\, e^{-f \cdot LET}$	-140.2	-132.2	-139.2	-133.2	-138.0	-132.0	-127.3
$c\, e^{-f \cdot LET} + g$	-134.6	-126.3	-133.2	-127.3	-132.0	-126.0	-121.3
$c \cdot e^{-f \cdot LET - h \cdot LET^2} + g$	-128.7	-128.3	-128.8	-122.8	-127.9	-121.9	-116.8
$(c + h \cdot LET) \cdot e^{-f \cdot LET} + g$	-128.6	-126.6	-128.5	-122.5	-127.3	-121.3	-116.5
$c \cdot ln(LET - h) \cdot e^{-f \cdot LET} + g$	-128.6	-125.4	-128.2	-122.2	-127.0	-119.8	-116.2
$\frac{c}{\Gamma(f \cdot LET + h + 1)} + g$	-128.7	-127.7	-128.7	-122.7	-127.7	-121.7	-116.7
$\frac{c\, \lambda^{f \cdot (LET - h)} \cdot e^{-\lambda}}{\Gamma(f \cdot (LET - h) + 1)} + g$	-122.7	-122.2	-122.8	-116.8	30.0	-115.9	-27.0

Supplemental Table 4: Bayesian Information Criteria (BIC) values determined by fitting the function create by combining the candidate slope and intercept functions to the training dataset's $D_{37\%}$ values. Each cell corresponds to the BIC value associated with a given slope and intercept function combination. Green cells indicate smaller BIC values (better performing functions) while red cells indicate larger BIC values (poorer performing functions).

Bayesian Information Criterion (BIC) value associated with the function predicting $D_{10\%}$							
Intercept / Slope	m	$p \cdot LET$	$m + p \cdot LET$	$m + p \cdot LET + q \cdot LET^2$	$q\, e^{s \cdot LET}$	$q\, e^{s \cdot LET} + m$	$q \cdot e^{s \cdot LET} + p \cdot LET + m$
$c\, e^{-f \cdot LET}$	-87.0	-79.0	-86.8	-80.8	-85.5	-79.5	-74.8
$c\, e^{-f \cdot LET} + g$	-81.3	-73.0	-80.8	-74.8	-79.5	-73.5	-68.8
$c \cdot e^{-f \cdot LET - h \cdot LET^2} + g$	-75.3	-75.2	-76.6	-70.7	-75.5	-69.5	-64.7
$(c + h \cdot LET) \cdot e^{-f \cdot LET} + g$	-75.3	-73.4	-76.2	-70.2	-74.8	-68.8	-64.2
$c \cdot ln(LET - h) \cdot e^{-f \cdot LET} + g$	-75.3	-72.2	-75.9	-69.9	-74.5	-68.5	-63.9
$\frac{c}{\Gamma(f \cdot LET + h + 1)} + g$	-75.3	-74.6	-76.5	-70.5	-75.2	-69.2	-64.5
$\frac{c\, \lambda^{f \cdot (LET - h)} \cdot e^{-\lambda}}{\Gamma(f \cdot (LET - h) + 1)} + g$	-69.3	-69.1	-70.6	-64.7	651.3	-63.5	46.9

Supplemental Table 5: Bayesian Information Criteria (BIC) values determined by fitting the function create by combining the candidate slope and intercept functions to the training dataset's $D_{50\%}$ values. Each cell corresponds to the BIC value associated with a given slope and intercept function combination. Green cells indicate smaller BIC values (better performing functions) while red cells indicate larger BIC values (poorer performing functions).

Bayesian Information Criterion (BIC) value associated with the function predicting $D_{10\%}$							
Intercept / Slope	m	$p \cdot LET$	$m + p \cdot LET$	$m + p \cdot LET + q \cdot LET^2$	$q\, e^{s \cdot LET}$	$q\, e^{s \cdot LET} + m$	$q \cdot e^{s \cdot LET} + p \cdot LET + m$
$c\, e^{-f \cdot LET}$	-27.4	-18.7	-26.9	-20.9	-25.7	-19.7	-14.9
$c\, e^{-f \cdot LET} + g$	-21.5	-12.7	-20.9	-14.9	-19.7	-13.7	-8.9
$c \cdot e^{-f \cdot LET - h \cdot LET^2} + g$	-15.5	-14.8	-16.9	-10.9	-15.7	-9.7	-4.9
$(c + h \cdot LET) \cdot e^{-f \cdot LET} + g$	-15.5	-13.0	-16.4	-10.4	-15.1	-9.1	-4.4
$c \cdot ln(LET - h) \cdot e^{-f \cdot LET} + g$	-15.5	-11.8	-16.1	-10.1	-14.7	-8.7	-7.4
$\frac{c}{\Gamma(f \cdot LET + h + 1)} + g$	-15.5	-14.2	-16.7	-10.7	-15.5	-9.5	-4.8
$\frac{c\, \lambda^{f \cdot (LET - h)} \cdot e^{-\lambda}}{\Gamma(f \cdot (LET - h) + 1)} + g$	-9.6	-8.7	-10.9	-4.9	167.5	-3.7	113.2

Supplemental Table 6: Bayesian Information Criteria (BIC) values determined by fitting the function create by combining the candidate slope and intercept functions to the training dataset's SF_{2Gy} values. Each cell corresponds to the BIC value associated with a given slope and intercept function combination. Green cells indicate smaller BIC values (better performing functions) while red cells indicate larger BIC values (poorer performing functions).

Bayesian Information Criterion (BIC) value associated with the function predicting $D_{10\%}$							
Intercept / Slope	m	$p \cdot LET$	$m + p \cdot LET$	$m + p \cdot LET + q \cdot LET^2$	$q\, e^{s \cdot LET}$	$q\, e^{s \cdot LET} + m$	$q \cdot e^{s \cdot LET} + p \cdot LET + m$
$c\, e^{-f \cdot LET}$	126.3	119.8	124.2	130.1	129.7	135.7	136.1
$c\, e^{-f \cdot LET} + g$	132.3	125.8	130.1	136.1	135.7	141.7	142.1
$c \cdot e^{-f \cdot LET - h \cdot LET^2} + g$	136.8	115.9	121.9	127.9	134.7	140.7	133.9
$(c + h \cdot LET) \cdot e^{-f \cdot LET} + g$	137.0	118.9	124.7	130.7	136.0	142.0	136.7
$c \cdot ln(LET - h) \cdot e^{-f \cdot LET} + g$	137.1	121.0	126.6	132.5	136.9	142.9	138.5
$\frac{c}{\Gamma(f \cdot LET + h + 1)} + g$	136.9	117.0	122.9	128.9	135.2	141.2	134.9
$\frac{c\, \lambda^{f \cdot (LET - h)} \cdot e^{-\lambda}}{\Gamma(f \cdot (LET - h) + 1)} + g$	142.8	122.1	128.0	134.0	243.3	146.8	228.1

Supplemental Note 4: Derivation of Model

Since our model predicts any arbitrary set of biological endpoints, $D_{SF,i}$, for a specified number of survival levels, SF_i, we can define a vector, **y**, whose i[th] element is the natural logarithm of the surviving faction corresponding to the ith endpoint, $D_{SF,i}$:

$$\boldsymbol{y} = \begin{bmatrix} \log(SF_1) \\ \log(SF_2) \\ \vdots \\ \log(SF_i) \end{bmatrix} \qquad (S1)$$

If the relationship between survival and dose can be described by the linear quadratic model, then we can define a matrix **D**:

$$\boldsymbol{D} = \begin{bmatrix} D_{SF,1} & D_{SF,1}^2 \\ D_{SF,2} & D_{SF,2}^2 \\ \vdots & \vdots \\ D_{SF,i} & D_{SF,i}^2 \end{bmatrix} \qquad (S2)$$

Which in turns allows us to write a vector expression describing the relationship between the predicted doses, $D_{SF,I}$, required to achieve the specified survival endpoints, SF_i, in terms of the linear quadratic parameters, $\boldsymbol{b} = -\begin{bmatrix} \alpha \\ \beta \end{bmatrix}$ as follows:

$$\boldsymbol{y} = \boldsymbol{D}\,\boldsymbol{b} \qquad (S3)$$

This expression can be rearranged to yield **b** that minimizes the sum-of-squares distance between **y** and **D b** as follows:

$$\left(\boldsymbol{D}^{\boldsymbol{T}}\boldsymbol{D}\right)^{-1}\boldsymbol{D}^{\boldsymbol{T}}\boldsymbol{y} = \boldsymbol{b} \qquad (S4)$$

$\boldsymbol{D}^{\boldsymbol{T}}\boldsymbol{y}$ is simple to evaluate in terms of the predicted endpoints:

$$\boldsymbol{D}^{\boldsymbol{T}}\boldsymbol{y} = \begin{bmatrix} D_{SF,1} & D_{SF,2} & \cdots & D_{SF,i} \\ D_{SF,1}^2 & D_{SF,2}^2 & \cdots & D_{SF,i}^2 \end{bmatrix} \begin{bmatrix} \log(SF_1) \\ \log(SF_2) \\ \vdots \\ \log(SF_i) \end{bmatrix} = \begin{bmatrix} \sum_{\boldsymbol{i}} D_{SF,i} \log(SF_i) \\ \sum_{\boldsymbol{i}} D_{SF,i}^2 \log(SF_i) \end{bmatrix} \qquad (S5)$$

$\left(\boldsymbol{D}^{\boldsymbol{T}}\boldsymbol{D}\right)^{-1}$ can also be easily computed since $\boldsymbol{D}^{\boldsymbol{T}}\boldsymbol{D}$ results in a 2×2 matrix:

$$\boldsymbol{D}^{\boldsymbol{T}}\boldsymbol{D} = \begin{bmatrix} D_{SF,1} & D_{SF,2} & \cdots & D_{SF,i} \\ D_{SF,1}^2 & D_{SF,2}^2 & \cdots & D_{SF,i}^2 \end{bmatrix} \begin{bmatrix} D_{SF,1} & D_{SF,1}^2 \\ D_{SF,2} & D_{SF,2}^2 \\ \vdots & \vdots \\ D_{SF,i} & D_{SF,i}^2 \end{bmatrix} = \begin{bmatrix} \sum_i D_{SF,i}\, D_{SF,i} & \sum_i D_{SF,i}\, D_{SF,i}^2 \\ \sum_i D_{SF,i}^2\, D_{SF,i} & \sum_i D_{SF,i}^2\, D_{SF,i}^2 \end{bmatrix} = \begin{bmatrix} \sum_i D_{SF,i}^2 & \sum_i D_{SF,i}^3 \\ \sum_i D_{SF,i}^3 & \sum_i D_{SF,i}^4 \end{bmatrix} \qquad (S6)$$

Being a 2×2 matrix, the inverse of $\boldsymbol{D^T D}$ can be computed easily via Cramer's rule:

$$\left(\boldsymbol{D^T D}\right)^{-1} = \frac{1}{\sum_i D_{SF,i}^2 \sum_i D_{SF,i}^4 - \sum_i D_{SF,i}^3 \sum_i D_{SF,i}^3} \begin{bmatrix} \sum_i D_{SF,i}^4 & -\sum_i D_{SF,i}^3 \\ -\sum_i D_{SF,i}^3 & \sum_i D_{SF,i}^2 \end{bmatrix} \quad (S7)$$

Consequently,

$$\left(\boldsymbol{D^T D}\right)^{-1} \boldsymbol{D^T y} = \frac{1}{\sum_i D_{SF,i}^2 \sum_i D_{SF,i}^4 - \sum_i D_{SF,i}^3 \sum_i D_{SF,i}^3} \begin{bmatrix} \sum_i D_{SF,i}^4 & -\sum_i D_{SF,i}^3 \\ -\sum_i D_{SF,i}^3 & \sum_i D_{SF,i}^2 \end{bmatrix} \begin{bmatrix} \sum_{\boldsymbol{i}} D_{SF,i} \log(SF_i) \\ \sum_{\boldsymbol{i}} D_{SF,i}^2 \log(SF_i) \end{bmatrix} =$$

$$\frac{1}{\sum_i D_{SF,i}^2 \sum_i D_{SF,i}^4 - \sum_i D_{SF,i}^3 \sum_i D_{SF,i}^3} \begin{bmatrix} \sum_i D_{SF,i}^4 \sum_{\boldsymbol{i}} D_{SF,i} \log(SF_i) - \sum_i D_{SF,i}^3 \sum_{\boldsymbol{i}} D_{SF,i}^2 \log(SF_i) \\ \sum_i D_{SF,i}^2 \sum_{\boldsymbol{i}} D_{SF,i}^2 \log(SF_i) - \sum_i D_{SF,i}^3 \sum_{\boldsymbol{i}} D_{SF,i} \log(SF_i) \end{bmatrix} = \boldsymbol{b} \quad (S8)$$

Thus, we have closed form expressions for the predicted α_{proton} and β_{proton} values:

$$\alpha_{proton} = \frac{\sum_i D_{SF,i}^4 \sum_{\boldsymbol{i}} D_{SF,i} \log(SF_i) - \sum_i D_{SF,i}^3 \sum_{\boldsymbol{i}} D_{SF,i}^2 \log(SF_i)}{\sum_i D_{SF,i}^3 \sum_i D_{SF,i}^3 - \sum_i D_{SF,i}^2 \sum_i D_{SF,i}^4} \quad (S9)$$

and

$$\beta_{proton} = \frac{\sum_i D_{SF,i}^2 \sum_{\boldsymbol{i}} D_{SF,i}^2 \log(SF_i) - \sum_i D_{SF,i}^3 \sum_{\boldsymbol{i}} D_{SF,i} \log(SF_i)}{\sum_i D_{SF,i}^3 \sum_i D_{SF,i}^3 - \sum_i D_{SF,i}^2 \sum_i D_{SF,i}^4} \quad (S10)$$

Where the $D_{SF,i}$ values for the specified number of survival levels, SF_i, are given by the expression:

$$D_{SF,i,proton} = c_i\, e^{-f_i * LET} \left(\frac{-\alpha_{xray} + \sqrt{\alpha_{xray}^2 - 4\,\beta_{xray} \cdot \ln(SF_i)}}{2\,\beta_{xray}} \right) + p_i \cdot LET \quad (S11)$$

Which comes from our observations of how the linear correlation between $D_{SF,I,proton}$ and $D_{SF,I,x\text{-}ray}$ varies with proton LET:

$$D_{SF,i,proton} = c_i\, e^{-f_i * LET} D_{SF,i,xray} + p_i \cdot LET \quad (S12)$$

And the fact that $D_{SF,x\text{-}ray}$ can be described in terms of $\alpha_{x\text{-}ray}$ and $\beta_{x\text{-}ray}$ by the linear quadratic model as:

$$D_{SF,i,xray} = \frac{-\alpha_{xray} + \sqrt{\alpha_{xray}^2 - 4\,\beta_{xray} \cdot \ln(SF_i)}}{2\,\beta_{xray}} \quad (S13)$$

Therefore, the expression for the predicted α_{proton} and β_{proton} values (Eqs. S9 and S10) are functions only of α_{xray}, β_{xray}, and the model parameters c_i, f_i, and p_i, corresponding to survival levels, SF_i. Note here, that this

construction does not require the use of any particular set of endpoints when predicting α_{proton} and β_{proton} and so the choice both of which endpoints to select, as well as how many to select, is arbitrary.

Supplemental Note 5: Choice of endpoints to incorporate in our model

Since the operator $\left(\boldsymbol{D}^T\boldsymbol{D}\right)^{-1}\boldsymbol{D}^T$ acting on **y** provides **b** that minimizes the sum-of-squares distance between **y** and **D b**, the use of additional endpoints will tend to provide **b** that more faithfully reproduces $\boldsymbol{y} = \boldsymbol{D}\,\boldsymbol{b}$. Consequently, given that the choice of endpoints to use in our formalism is arbitrary, we must decide which, and how many endpoints to use in our final parameterization. To do so, we must weigh how much more faithfully the model predicts the data using these additional parameters against the informational cost of including those extra parameters. To do this, we calculated the BIC associated with the different possible parameterizations of our model as follows:

i) For each of $D_{5\%}$, $D_{10\%}$, $D_{20\%}$, $D_{37\%}$, $D_{50\%}$ and SF_{2Gy}, we calculated the free parameters c_i, f_i, and p_i, associated with those endpoints

ii) Then for each possible combination of endpoints, we predicted the α_{proton} and β_{proton} values of the whole database using Eqs. S9 and S10.

iii) We then calculated the relative distance between the predicted curves and the measured curves using the L2 norm normalized by the mean inactivation dose as follows:

$$\frac{\int_0^\infty \left|SF_{predicted}(D) - SF_{measured}(D)\right|^2 dD}{\int_0^\infty \left|SF_{predicted}(D)\right|^2 dD} \qquad (S14)$$

This metric was chosen since it incorporates information across the whole survival curve, and not being restricted to a particular dose or survival level, gives a description of how the models perform overall.

iv) We then defined the maximum log-likelihood function, ln(L), in terms of the sum over the L2 norms of all the data (N datapoints) in the training dataset[24]:

$$\ln(L) = -\frac{N}{2}\left[\ln(2\,\pi) + 1 - \ln(N) + \ln\left(\sum_i \frac{\int_0^\infty \left|SF_{predicted,i}(D) - SF_{measured,i}(D)\right|^2 dD}{\int_0^\infty \left|SF_{predicted,i}(D)\right|^2 dD}\right)\right] \qquad (S15)$$

v) Then, for each parameterization, we calculated the BIC, where p is the number of parameters and n is number of survival curves in the training dataset, as:

$$BIC = p\ln(n) - 2\ln(L) \qquad (S16)$$

vi) We then compared the BIC values and selected the parameterization which minimized the BIC values

vii) We also repeated this process using different parameterizations of the intercept, allowing its LET dependence to be described either by a constant, a linear function, a linear function with no vertical offset, or an exponential function with no vertical offset to clarify which parameterization, when used across multiple endpoints, yields the best description of α_{proton} and β_{proton}.

Supplemental Note 6: Parameterizations used for Wedenberg, McNamara and Mairani Models

In assessing the goodness of fit of the previously published models (Table 6 and Supplemental Note 5), we retrained them on our training dataset. In many of the original publications detailing these empirical models, a variety of LET-dependent functions were assessed to determine which function best predicted the training data. In some cases, we noted that the best parameterizations based on the goodness-of-fit metrics we calculated were different from those in the original publications or depended on the goodness-of-fit metric used. For example, the function that minimized the χ^2/v for the Mairani et al. model[4] was their *fLQC* (linear-quadratic-cubic) function, and the function that minimized the BIC value was their *fL (*linear*)* function. This discrepancy arises largely because the BIC includes a larger penalty for the number of parameters used in the fit, thus favoring the less complicated linear function. In cases such as this, where different parameterizations could be justified among the previously published models, to give as much credit to these models as possible, we conservatively chose whichever parameterization minimized the goodness-of-fit metric investigated. Below we summarize the actual parameterizations of those models we used in this work.

For Wedenberg et al model, with free parameter a_1, proton dose D, and LET L the function selected was:

$$RBE_{Wedenberg} = -\frac{1}{2D}\left(\frac{\alpha}{\beta}\right)_{xray} + \frac{1}{D}\sqrt{\frac{1}{4}\left(\frac{\alpha}{\beta}\right)^2_{xray} + \left(a_1 L + \left(\frac{\alpha}{\beta}\right)_{xray}\right) D + D^2}$$

For McNamara et al model with free parameters a_1, a_2, a_3 and a_4, proton dose D and LET L, the function selected was:

$$RBE_{McNamara} = \frac{1}{2D}\left(\sqrt{\left(\frac{\alpha}{\beta}\right)^2_{xray} + 4D\left(\frac{\alpha}{\beta}\right)_{xray}\left(a_1 + a_2\frac{L}{\left(\frac{\alpha}{\beta}\right)_{xray}}\right) + 4D^2\left(a_3 + a_4 L\sqrt{\left(\frac{\alpha}{\beta}\right)_{xray}}\right)} - \left(\frac{\alpha}{\beta}\right)_{xray}\right)$$

For Mairani et al model when minimizing the RRS for the predictions of the RBE at 2 Gy, we used the following function with free parameters a_1, a_2, a_3 and a_4, proton dose, D and LET, L:

$$RBE_{Mairani} = -\frac{1}{2D}\left(\frac{\alpha}{\beta}\right)_{xray} + \frac{1}{D}\sqrt{\frac{1}{4}\left(\frac{\alpha}{\beta}\right)^2_{xray} + D\left(\frac{\alpha}{\beta}\right)_{xray}\left(1 + \left[a_1 + \left(\frac{\beta}{\alpha}\right)_{xray}\right] f_{LQC}\right) + D^2\left(a_2 e^{\left[-\left(\frac{L-a_3}{a_4}\right)^2\right]}\right)}$$

Where f_{LQC} is a function of three additional free parameters, a_5, a_6 and a_7, and the LET, L:

$$f_{LQC} = a_5 L + a_6 L^2 + a_7 L^3$$

For Mairani et al model when minimizing the BIC associated with the L2 norm, we used the following function with free parameters a_1, a_2, a_3 and a_4, proton dose, D and LET, L:

$$RBE_{Mairani} = -\frac{1}{2D}\left(\frac{\alpha}{\beta}\right)_{xray} + \frac{1}{D}\sqrt{\frac{1}{4}\left(\frac{\alpha}{\beta}\right)^2_{xray} + D\left(\frac{\alpha}{\beta}\right)_{xray}\left(1+\left[a_1+\left(\frac{\beta}{\alpha}\right)_{xray}\right]f_L\right) + D^2\left(a_2 e^{\left[-\left(\frac{L-a_3}{a_4}\right)^2\right]}\right)}$$

Where f_L is a function of one additional free parameter, a_5, and the LET, L:

$$f_L = a_5 L$$

The values of the free parameters found for these models are summarized in Supplemental Table 7-Supplemental Table 9 below. For reference, Supplemental Table 10 contains the original parameterizations and parameter values reported in the original publications of the Wedenberg, McNamara, and Mairani models.

Supplemental Table 7: Model parameters found for the Wedenberg et al., McNamara et al., Mairani et al., fit to the training dataset, including only data with LET values <=37.8 keV/μm.

**Model**	**Minimization**	**Model Parameters**						
		**a_1**	**a_2**	**a_3**	**a_4**	**a_5**	**a_6**	**a_7**
**Wedenberg et al**	RSS for RBE @ 2Gy	4.8898 E-01						
	L2 norm	3.1293 E-01						
**McNamara et al**	RSS for RBE @ 2Gy	9.6231 E-01	2.5786 E-01	1.0633 E+00	1.8793 E-02			
	L2 norm	9.2328 E-01	3.5049 E-01	1.0113 E+00	-1.9297 E-03			
**Mairani et al**	RSS for RBE @ 2Gy	5.8612 E-02	1.1496 E+00	-5.3320 E-02	3.7951 E+00	5.2266 E-01	1.9482 E-03	-1.5228 E-04
	L2 norm	1.6529 E-03	1.2964 E+00	-2.5077 E+02	4.5653 E+02	3.2333 E-01		

Supplemental Table 8: Model parameters found for the Wedenberg et al., McNamara et al., Mairani et al., fit to the training dataset, including only data with LET values <=30 keV/μm.

Model	Minimization	Model Parameters						
		a_1	a_2	a_3	a_4	a_5	a_6	a_7
**Wedenberg et al**	RSS for RBE @ 2Gy	4.7889 E-01						
	L2 norm	3.0744 E-01						
**McNamara et al**	RSS for RBE @ 2Gy	9.8184 E-01	3.0878 E-01	1.0334 E+00	1.5660 E-02			
	L2 norm	9.1502 E-01	3.4365 E-01	1.0111 E+00	-3.2273 E-04			
**Mairani et al**	RSS for RBE @ 2Gy	4.9050 E-02	1.1358 E+00	1.1557 E-01	3.6449 E+00	5.1573 E-01	8.4542 E-03	-4.3381 E-04
	L2 norm	-6.6390 E-03	4.9811 E+03	1.3622 E+03	4.6519 E+02	3.0592 E-01		

Supplemental Table 9: Model parameters found for the Wedenberg et al., McNamara et al., Mairani et al., fit to the training dataset, including only data with LET values <=20 keV/μm.

Model	Minimization	Model Parameters						
		a_1	a_2	a_3	a_4	a_5	a_6	a_7
**Wedenberg et al**	RSS for RBE @ 2Gy	4.8112 E-01						
	L2 norm	2.6565 E-01						
**McNamara et al**	RSS for RBE @ 2Gy	9.1371 E-01	2.7225 E-01	1.0255 E+00	3.5893 E-03			
	L2 norm	9.5866 E-01	2.9407 E-01	1.0356 E+00	1.9813 E-02			
**Mairani et al**	RSS for RBE @ 2Gy	6.2763 E-02	1.4858 E+00	1.9062 E+00	4.4841 E+00	-2.2423 E-01	1.1921 E-01	-4.3369 E-03
	L2 norm	-2.5810 E-02	5.9378 E+06	7.8717 E+02	1.9900 E+02	2.1467 E-01		

Supplemental Table 10: Model parameterization and parameters for the Wedenberg et al., McNamara et al., Mairani et al., models as reported in the original publications.

Model	Parameterization	Model Parameters			
	$\boldsymbol{RBE}\left(\boldsymbol{D},\boldsymbol{L},\left(\frac{\boldsymbol{\alpha}}{\boldsymbol{\beta}}\right)_{\boldsymbol{xray}}\right)=$	**a1**	**a2**	**a3**	**a4**
**Wedenberg et al**	$-\frac{1}{2D}\left(\frac{\alpha}{\beta}\right)_{xray}+\cdots$ $+\frac{1}{D}\sqrt{\frac{1}{4}\left(\frac{\alpha}{\beta}\right)^2_{xray}+\left(a_1L+\left(\frac{\alpha}{\beta}\right)_{xray}\right)D+D^2}$	4.34 E-01			
**McNamara et al**	$\frac{1}{2D}\left(\sqrt{\left(\frac{\alpha}{\beta}\right)^2_{xray}+4D\left(\frac{\alpha}{\beta}\right)_{xray}\left(a_1+a_2\frac{L}{\left(\frac{\alpha}{\beta}\right)_{xray}}\right)+\cdots+4D^2\left(a_3+a_4L\sqrt{\left(\frac{\alpha}{\beta}\right)_{xray}}\right)}-\left(\frac{\alpha}{\beta}\right)_{xray}\right)$	9.9906 4 E-01	3.5605 4 E-01	1.1012 9 E+00	3.8703 E-02
**Mairani et al**	$-\frac{1}{2D}\left(\frac{\alpha}{\beta}\right)_{xray}+\cdots$ $+\frac{1}{D}\sqrt{\frac{1}{4}\left(\frac{\alpha}{\beta}\right)^2_{xray}+D\left(\frac{\alpha}{\beta}\right)_{xray}\left(1+\left(\frac{\beta}{\alpha}\right)_{xray}a_1\right)+\cdots \ldots+D^2\left(a_2e^{\left[-\left(\frac{L-a_3}{a_4}\right)^2\right]}\right)}$	3.77 E-01	1.000 E+00	3.28 E+00	2.79 E+01

Supplemental Note 7: Goodness-of-Fit Metric Values for different high LET filters

In this work, we included survival data in our training dataset up to LET values of 37.8 keV/μm, similar to Mairani et al., however the works by McNamara et al. and Wedenberg et al. only incorporated data up to LET values of 20 and 30 keV/μm, respectively. For completeness, we recalculated the goodness-of-fit metrics after imposing the same LET criteria on the database and retraining the models accordingly (Supplemental Table 12 and Supplemental Table 13), as well as without excluding any high LET data (Supplemental Table 11).

Supplemental Table 11: Goodness-of-Fit metrics for different models fit to our training dataset, minimizing either the RBE at a proton dose of 2 Gy or the L2 norm between the predicted and measured survival curves across all LET values up to 88.8 keV/μm.

Model	χ^2/v (RBE_{2Gy})	BIC (RBE_{2Gy})	BIC (L2 norm)
Wedenberg et al	0.0951	374.10	-602.11
McNamara et al	0.0880	371.83	-601.05
Mairani et al	0.0720	268.64	-597.60
Our Model	0.0690	239.98	-737.51

Supplemental Table 12: Goodness-of-Fit metrics for different models fit to our training dataset, but only including data for LET values <= 30 keV/μm, similar to Wedenberg et al., and by minimizing either the RBE at a proton dose of 2 Gy or the L2 norm between the predicted and measured survival curves across the database.

Model	χ^2/v (RBE_{2Gy})	BIC (RBE_{2Gy})	BIC (L2 norm)
Wedenberg et al	0.0758	212.88	-716.81
McNamara et al	0.0716	215.04	-706.10
Mairani et al	0.0690	222.36	-693.93
Our Model	0.0673	213.81	-727.58

Supplemental Table 13: Goodness-of-Fit metrics for different models fit to the filtered database, only including LET values up to 20 keV/um similar to McNamara et al., and by minimizing either the RBE at a proton dose of 2 Gy or the L2 norm between the predicted and measured survival curves across the database.

Model	χ^2/v (RBE_{2Gy})	BIC (RBE_{2Gy})	BIC (L2 norm)
**Wedenberg et al**	0.0715	165.00	-710.26
**McNamara et al**	0.0680	172.21	-699.39
**Mairani et al**	0.0649	174.53	-691.29
**Our Model**	0.0640	168.85	-733.60

Regardless of how the higher LET data are handled, our model provides the lowest χ^2/v when fitting to the RBE at the 2 Gy dose level, and the smallest BIC values when minimizing the L2 norms. However, when the higher LET data are excluded, the Wedenberg model provides a slightly smaller BIC when minimizing the RSS around the RBE_{2Gy} values. It is important to note here though that this that this does not suggest that the Wedenberg model is the most accurate around the 2 Gy dose level – our model is consistently the most accurate – but rather that when the higher LET data are not included, and one only wishes to predict the RBE at a single dose-level, 2 Gy, then the gain in accuracy our model (as well as those by McNamara and Mairani) provide may not be warranted given the use of the additional free parameters. However, given that the BIC associated with the L2 norms are considerably better for our model than any of the other models, regardless of the LET range investigated suggests that our model is the most robust across dose levels. It is also worth noting that even when the very high LET data are included, our model doesn't see such a drastic change in performance as the other models, further implying that our model is much more robust across a wide range of proton LET values.

Supplemental Note 8: Linear correlations between proton and x-ray sensitivity for the parameters D5%, D20% and SF2Gy

Fig. 1 shows the linear correlations between proton and x-ray radiosensitivity for the parameters $D_{10\%}$, $D_{37\%}$ and $D_{50\%}$. Similar trends can be seen for the parameters $D_{5\%}$, $D_{20\%}$ and SF_{2Gy} as shown in Supplemental Figure 1 below.

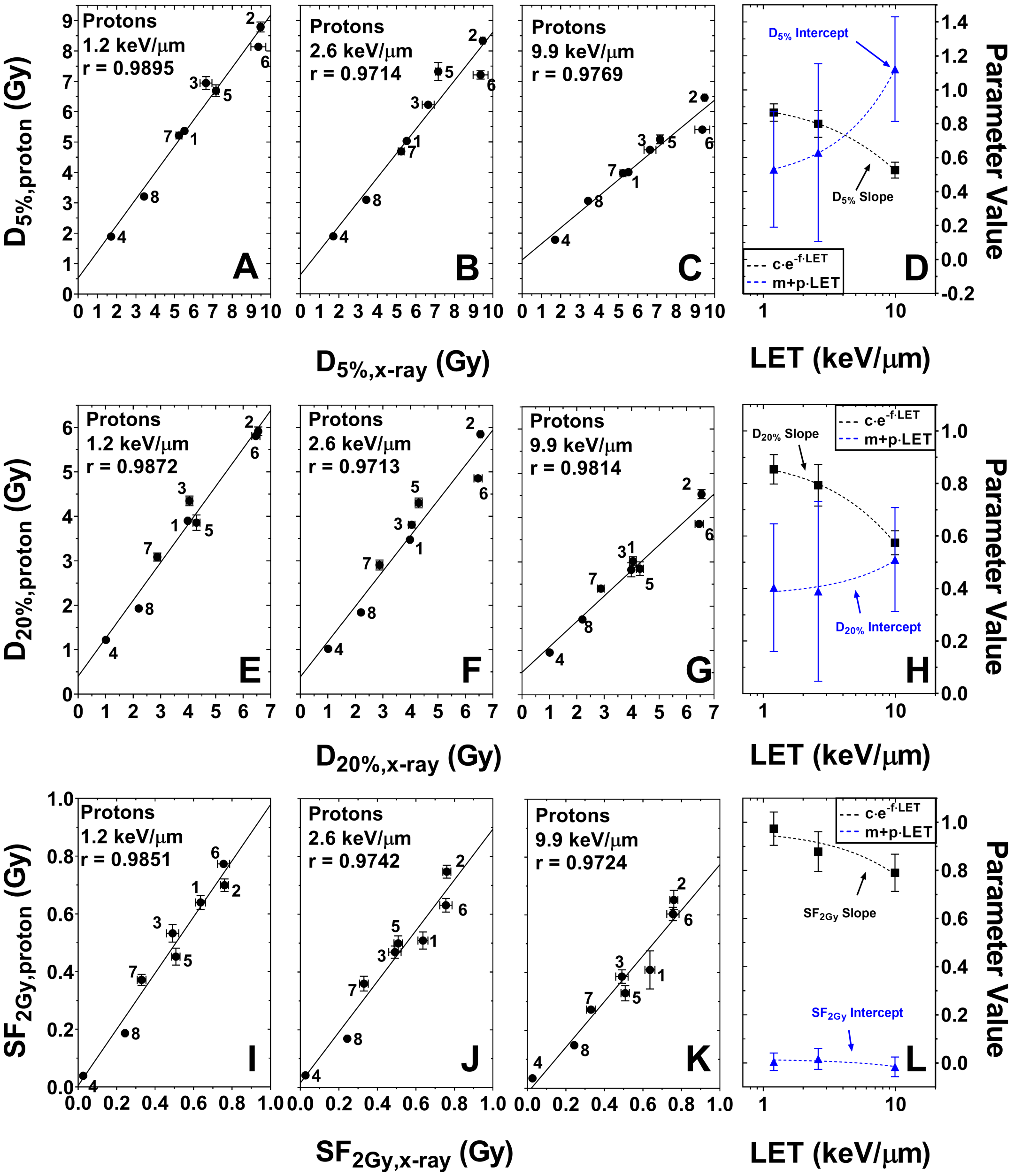

Supplemental Figure 1: Linear correlation between proton and x-ray radiosensitivity for the radiosensitivity, parameterized by the dose required to reduce cell survival to 5%, $D_{5\%}$ (A-C), and 20%, $D_{20\%}$ (D-F), and the surviving fraction after a dose of 2 Gy, SF_{2Gy}, (G-I), for cells exposed to 6 MV x-rays and protons with dose-weighted LET values of 1.2 keV/μm (A,D,G), 2.6 keV/μm (B, E, H) or 9.9 keV/μm (C, F, I). Numbers indicate the cell lines [1: H460, 2: H1299, 3: M059K, 4:M059J, 5:BxPC3, 6:HT1080, 7:HT1080-shRad51[IND], 8: HT1080-shDNAPKcs.The trends for other radiosensitivity parameters are given in Fig. 1.

Supplemental Note 9: Accuracy of our model

We performed leave one out cross-validation to quantify the predictive accuracy of our model. After predicting each data point in the training dataset set in this way, we bootstrapped the percent deviations in the predictions to determine the prediction intervals associated with our model. These prediction intervals are given in Supplemental Table 14. Generally, our model performs better at higher doses (lower survival levels), but generally the accuracy is on the order of 15-30%.

Supplemental Table 14: Our model's prediction intervals for the radiosensitivity parameters $D_{10\%}$, $D_{50\%}$ and SF_{2Gy}, and the RBE for at 10% and 50% survival levels, as well as at the 0.5Gy. 1Gy, 2Gy and 5 Gy dose levels. We also show the value of the square root of the L2 norm relative to $\bar{D}$, the integral of the survival curve, to quantify the general accuracy of our model's predictions across all dose and survival levels.

Parameter	68.3% Prediction Interval	95% Prediction Interval
$D_{10\%}$	[-2.7, 21.0]%	[-26.2, 47.7]%
$D_{50\%}$	[-16.3, 23.8]%	[-50.6, 44.2]%
SF_{2Gy}	[-14.5, 23.2]%	[-40.8, 66.8]%
$RBE_{10\%}$	[-26.0, 3.1]%	[-92.7, 20.6]%
$RBE_{50\%}$	[-30.5, 14.3]%	[-79.6, 33.6]%
$RBE_{@0.5Gy}$	[-35.8, 37.8]%	[-73.4, 63.5]%
$RBE_{@1Gy}$	[-32.1, 29.8]%	[-64.0, 51.9]%
$RBE_{@2Gy}$	[-27.5, 18.6]%	[-65.8, 36.2]%
$RBE_{@5Gy}$	[-23.2, 4.8]%	[-74.8, 21.0]%
$L2/\bar{D}$	[2.1, 12.2]%	[0.7, 38.6]%
